# Assessment of Lead in Drinking Water from Multiple Drinking Water Sampling Programs for a Midsize City

**DOI:** 10.1021/acs.est.2c06614

**Published:** 2022-12-23

**Authors:** Vasikan Vijayashanthar, Mitchell J. Small, Jeanne M. VanBriesen

**Affiliations:** † Department of Civil and Environmental Engineering, Carnegie Mellon University, 5000 Forbes Avenue, Pittsburgh, Pennsylvania 15213, United States; ‡ Department of Engineering and Public Policy, Carnegie Mellon University, 5000 Forbes Avenue, Pittsburgh, Pennsylvania 15213, United States

**Keywords:** Lead, Drinking water, LCR compliance, Corrosion control, Lead and copper rule

## Abstract

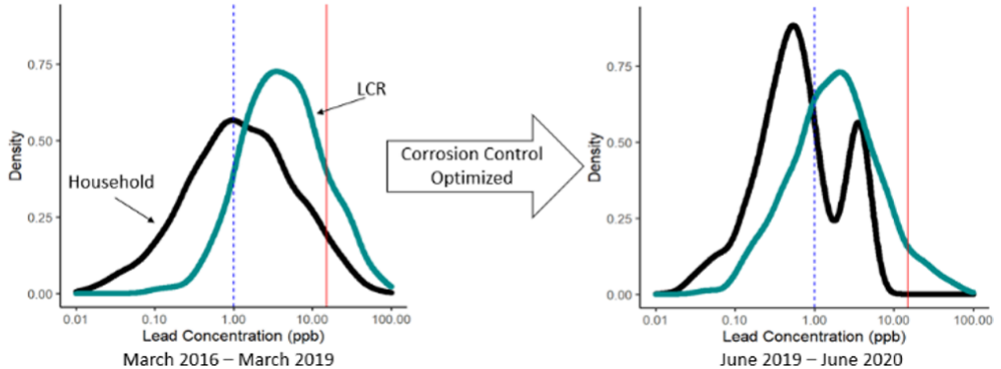

Following an exceedance of the lead action level for drinking water in 2016, the Pittsburgh Water and Sewer Authority (PWSA) undertook two sampling programs: the required biannual Lead and Copper Rule (LCR) compliance testing and a home sampling program based on customer requests. The LCR sampling results, at locations expected to be elevated when corrosion is not well controlled, had higher concentrations than customer-requested homes, with 90th percentile values for the LCR sites exceeding the action level through 2019 (except for June 2018). Customer-requested concentrations showed greater variability, with the median lead concentration for customer-requested samples below detection for each year of sampling, suggesting only some homes show elevated lead when corrosion control is not fully effective. Corrosion control adjustments brought the utility back into compliance in 2020 (LCR 90th percentile of 5.1 ppb in June 2020); customer-requested sampling after the addition of orthophosphate indicated below detection levels for 59% of samples. Monte Carlo simulations indicate LCR samples do not all represent high lead risk sites, and the application of corrosion control more significantly affects higher lead concentration sites. Broader water quality sampling provides information about specific homes but is not well suited to assessing the efficacy of corrosion control efforts by utilities.

## Abstract 2

Different distributions of residential drinking water lead concentrations result from different sampling approaches and responses to corrosion control adjustments.

## 1 Introduction

Lead-containing materials were widely used historically in components of the drinking water distribution network leading to customer homes (“service lines”) and in indoor pipes, fixtures, and solder. The corrosion of these materials can result in increased lead concentrations in drinking waters at homes, schools, and other locations.^1^ Exposure to lead in childhood is associated with attention deficit, aggression, hyperactivity, and decreased IQ.^2,3^ Adult exposure is associated with increased blood pressure, kidney disease, and neurological effects similar to those resulting from childhood exposure.^4^ There is no safe concentration of lead in drinking water.^5^ The lead water crises in Washington, DC, in 2000 and in Flint, Michigan, in 2014 highlighted how the nation’s aging water infrastructure, coupled with inadequate decision support and management, can result in significant public health risk.^6,7^

To reduce exposure to lead in drinking water, the Lead and Copper Rule (LCR)^8^ requires public water systems (PWS) to apply chemical corrosion control with limited exceptions (see Supporting Information (SI) Section I for additional details). Corrosion control chemicals can include those added for corrosion inhibition (e.g., phosphates) and those used for pH, alkalinity, or hardness adjustment. Corrosion control chemicals cause deposition of a precipitate layer over lead plumbing, preventing or reducing release of lead into the water. Utilities monitor the efficacy of corrosion control by testing for lead and copper concentrations in water from consumers’ taps. The LCR requires samples to be collected at a specific number of single-family homes with known lead service lines (LSL) or suspected indoor lead plumbing/solder (i.e., 100 sites for systems with greater than 100,000 connections). These sites are called Tier I and are expected to represent worst case conditions for the negative effects of corrosion on lead concentrations.^8,9^ Homes that do not contain lead in plumbing or solder are expected to have lower concentrations even if corrosion control was ineffective and are not sampled to assess the efficacy of corrosion control. Thus, Tier I sites are not intended to represent lead concentrations throughout the system, since many homes may have no lead plumbing. Corrosion control is considered adequate if fewer than 10% of the tested Tier I homes have lead concentrations that exceed 15 parts per billion (ppb). Action to improve corrosion control is required if the 90th percentile value of the LCR-tested Tier I sites exceeds 15 ppb (the action level, AL).^8^

Even though LCR sampling is intended to monitor corrosion control efficacy, consumers may interpret results as indicative of drinking water exposure or risk.^10^ In the absence of extensive home-level water testing, the relationship between LCR-based results and broader drinking water lead concentrations cannot be determined. Some cities with recent lead in drinking water problems, like Flint, Michigan, have established extensive residential sampling programs to allow concerned consumers the opportunity to test their home faucets.^11^ Results from this type of program are subject to self-selection bias, but nonetheless provide additional information about the distribution of lead concentrations in water within a community.^12,13^

In 2016, the public water utility serving most of the city of Pittsburgh, Pennsylvania, exceeded the federal AL despite the use of caustic soda and pH adjustment for corrosion control.^14^ LCR samples taken in summer 2016 had a 90th percentile value of 22 ppb, exceeding the AL. Previous results in 2013, 2010, and 2007 had 90th percentile values below the AL (14.8, 10.4, and 9.0 ppb, respectively).^14^ Following an exceedance of the federal AL, the LCR requires compliance sampling to be performed every six months (at Tier I sites) as the utility continues remediation and public education actions. These additional samples and increased frequency are intended to monitor the performance of corrosion control, which may be modified by the utility in order to return to compliance. In addition to the required Tier I sampling, as part of the community response and public education campaign, the utility also offered a customer-requested lead sampling and testing program.^15^ These samples resulted in a data set that contained many sites that would not be included in LCR sampling because they did not have lead service lines or indoor lead plumbing.

The present study evaluates system-wide lead in water concentrations based on the extensive customer-requested sampling data during the LCR exceedance period (2016 to 2019) and after the return to compliance (in 2020). The customer-requested data and the LCR data are compared to evaluate the use of different sampling plans for assessment of corrosion control. Considering sampling results from before and after corrosion control changes in early 2019 further contributes to understanding how sampling choices affect information gained on the performance of corrosion control.

## 2 Materials and Methods

### 2.1 Study Location and Time Period

The Pittsburgh Water and Sewer Authority (PWSA) provides water to approximately 300,000 customers throughout the City of Pittsburgh, producing on average 265 million liters of water per day. Much of the distribution system was built around the 1920s and serves most of the city (see Figure S1 in the Supporting Information).

**Figure 1:**
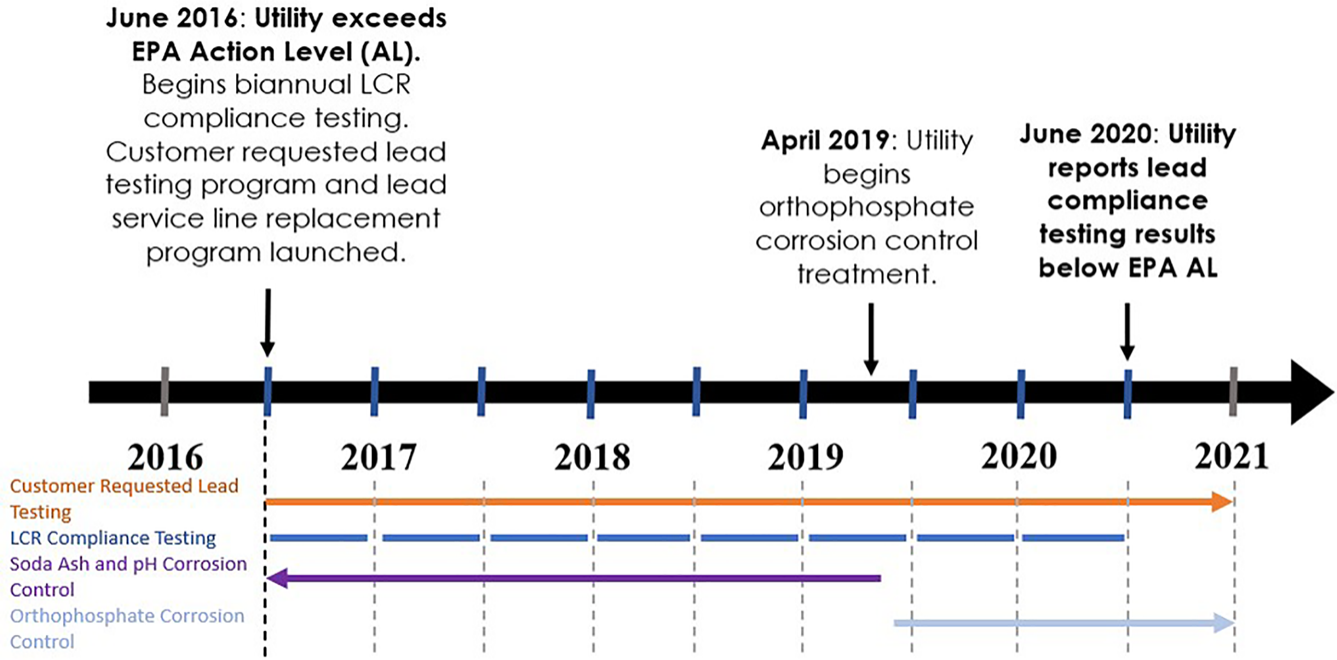
Timeline of lead compliance events and actions. Customer-requested lead testing data were collected from February 2016 through June 2020. LCR samples were collected during the first half of the year and reported in June and during the second half of the year and reported in December, from June 2016 through June 2020. Orthophosphate dosing began in April 2019.

The present study focuses on Pittsburgh, PA, between 2016 and 2020 as this represents a time period when the utility was working to improve corrosion control (after reporting an exceedance) and to take other corrective measures required by the LCR. Figure 1 provides a timeline of these events and actions by the utility.

### 2.2 Drinking Water Data

Table 1 summarizes the drinking water lead sampling data used for the present analyses, including the data source, the time period for sample collection, the total number of samples collected through each program, and the number of samples below the reporting limit (BRL). The last two columns provide information on how the data were grouped for analyses.

**Table 1:** Description of Lead Sampling Data Available from LCR Testing and Customer-Requested Testing

Data Source	Data Description	Collection Periods	Number of Samples (No. BRL)	Annual Grouping	Treatment Grouping
LCR compliance sampling reported every six months from mid-2016 through mid-2020	Lead concentrations in residential samples at Tier I sites selected for LCR monitoring	June and December 2016, 2017, 2018, 2019; June only 2020 (recorded as reporting month and year, e.g., June 2016 for samples analyzed in the first half of the year)	Total −1275 (435); June 2016 – 100 (45); December 2016 – 159 (52); June 2017 – 128 (46); December 2017 – 118 (38); June 2018 – 106 (57); December 2018 – 161 (68); June 2019 – 177 (39); December 2019 – 168 (65); June 2020 – 158 (63)	2016 = June 2016 and December 2016; 2017 = June 2017 and December 2017; 2018 = June 2018 and December 2018; 2019 = June 2019 and December 2019; 2020 = June 2020	Pre-Orthophosphate = June 2016, Dec 2016, June 2017, Dec 2017, June 2018, Dec 2018; Post-Orthophosphate = Dec 2019, June 2020; Excluded = June 2019
Customer-requested lead sampling results	Lead concentrations in residential samples at customer-requested locations	2016–2020 (collection date not indicated; analysis date provided)	Total – 14324 (9512); 2016 – 2901 (1623); 2017 – 6327 (4617); 2018 – 2309 (1753); 2019 – 2410 (1294); 2020 – 347 (225)	Based on sample analysis date: 2016 = 2/15/16 to 12/31/16; 2017 = 1/1/17 to 12/31/17; 2018 = 1/1/18 to 12/31/18; 2019 = 1/1/19 to 12/31/19; 2020 = 1/1/20 to 6/29/20	Pre-Orthophosphate = March 2016–March 2019; Post-Orthophosphate = June 2019–June 2020; Excluded = April–May 2019

The collection of LCR samples was from utility-identified Tier I homes (with known LSLs, indoor lead plumbing, or copper plumbing with lead solder). In the data source, the locations of these sites are redacted to street name and street block number, and no date of analysis or date of sample collection was available. Residents collect the water as a first-draw one-liter sample after a 6 h stagnation period, and they mail this sample to the utility for analysis. Results of LCR sampling are reported biannually in June and December, from June 2016 to June 2020. Data from the LCR sampling are publicly available on the utility’s lead response Web site.^16^

In addition to the increased frequency of required compliance sampling following the LCR exceedance, the utility also initiated a no-cost customer-requested lead sampling and testing program. The program enabled consumers to request a lead sampling kit and send a self-collected first-draw sample to the utility. To maintain consumer privacy, the location information from the customer-requested drinking water testing program was anonymized (to block level on each street).^17^ Data from this program, which included over 14,000 samples since 2016, are publicly available for download on the utility’s lead response Web site.^16^ Each customer-requested data point consists of the street name, street block number, and the ZIP code from where it was collected; the lead concentration measured for the sample; and the date of sample analysis (but not the date of sample collection).

### 2.3 Statistical Analyses

Within each data set, for samples that were reported by the laboratory to have lead concentrations that were “below the reporting limit” or “nondetect”, a semiparametric log-normal regression on order statistics (ROS) method was used for imputation.^18^ Between 2016 and 2020, PWSA used multiple laboratories for lead testing, and these laboratories had varying reporting limits (from 1 to 4 ppb). Each sample reported as below detection was imputed relative to the reporting limit for that sample (see SI Section II for more information).

For each data type, sampling period, or data grouping (e.g., annual), the data were log transformed and fit to a normal distribution. Data were grouped by year for preliminary analysis as shown in Table 1. LCR data that were reported in June and December (other than 2020 with samples only reported June) were aggregated by corresponding year. Customer-requested data that were recorded by sample analysis date were aggregated by year; however, in this case, it is possible that samples collected in December were analyzed in January. SI Section III presents details of the assessment of goodness of fit for the distribution for each data grouping.

In several cases, statistical tests indicate that single distributions do not appear to represent the customer-requested data adequately (e.g., Figure S8 for the 2016 results). The use of mixture distributions, designed to represent different groups of data, was assessed for each year and for pre- and post-orthophosphate introduction.^19^Figures S13 to S18 show the mixture modeling fits. Goodness of fit measures including Kolmogorov–Smirnov tests, and Bayesian information criterion (BIC) statistics were computed for the mixture distributions (see Table S12). When evaluated quantitatively, these goodness of fit tests generally assume independent observations. Like many real-world data sets, this assumption would be invalidated due to temporal or spatial correlation or the presence of common explanatory variables among sampled homes. As such, the quantitative goodness of fit results presented here should be interpreted with care, indicative of broadscale, qualitative agreement or differences in the samples, rather than precise confidence intervals or p-values for the corresponding populations.

#### System-Wide Lead Concentration Assessment: Customer-Requested Lead Concentrations

First draw samples collected in customer-requested homes are analyzed. Pairwise statistical tests of similarity in the distribution (Kolmogorov–Smirnov test) and mean rank (Wilcoxon rank sum test) were conducted to determine if the sample distributions are similar year over year (see Tables S13 and S14).

#### System-Wide Corrosion Control Assessment: Customer-Requested and LCR Concentrations

Customer-requested lead sampling grouped by year were compared to annually grouped LCR sampling. Statistical tests of distribution (Kolmogorov–Smirnov test) and mean rank (Wilcoxon rank sum test) similarity were performed (see Tables S15 and S16).

#### Pre- and Post-Corrosion Control Adjustment

Data from the customer-requested sampling and LCR sampling were analyzed for statistically significant changes in lead concentrations after the introduction of orthophosphate in 2019, which was expected to improve corrosion control. It is important to note that the optimization of orthophosphate dosing can take years, and lead concentrations can continue to decrease after dose optimization.^9,20,21^ LCR samples and customer-requested samples after the introduction of orthophosphate were expected to be lower, and the 90th percentile concentrations in the LCR data would be expected to be below the EPA action level once corrosion control was optimized. Table 1, far right column, describes how the available data from each data source were divided into pre- and post-orthophosphate introduction data sets. Some data were omitted in this analysis as the timing of the samples could not be confirmed. LCR data reported in June 2019 (collected between January and May of 2019) were omitted as were customer-requested data with analysis dates between April and May 2019. SI Section III.E provides more information on distribution fitting for the pre- and post-orthophosphate data.

A comparison of the two-component mixture model of the customer-requested data and the log-normal distributions fit to the LCR data, pre- and post-orthophosphate introduction, was performed to determine if there were similarities in the distribution of lead concentrations for the two component distributions and the LCR data that might suggest whether the customer-requested data include some homes that are similar to LCR Tier I locations and other homes that are not.

#### Monte Carlo Simulation

Monte Carlo sampling of the pre- and post-orthophosphate customer-requested mixture models was performed to determine if the customer-requested data provide similar information about the utility’s LCR compliance status as the LCR sampling does. Here, 150 samples (similar to the number of LCR samples reported by the utility per compliance sampling period) were drawn 10,000 times from the fitted pre- and post-orthophosphate customer-requested data distributions that represent years the utility was out of compliance (March 2016–March 2019) and when it returned to compliance (June 2019–June 2020). Further details on this are provided in SI Section III.F. The 90th percentile was then calculated for each of these 10,000 sample sets using the “count-up” method specified in the LCR.^8^ A comparison was conducted of the reported 90th percentile from the LCR data and the calculated 90th percentiles from the sampled customer-requested data. This analysis was repeated for each year of the customer-requested data as well.

#### Customer-Requested Sampling Bias Assessment

The block-lot location information for customer-requested data was geolocated using ArcGIS, and an assessment of sampling bias was performed to determine if areas in the city with lead sample concentrations above the detection limit exhibited higher sampling frequency than areas with fewer samples above the detection limit. The customer-requested data were aggregated to the ZIP code level for this analysis. Housing information for each ZIP code was retrieved from the U.S. Census Web site. Further details are provided in SI Section III.G.

## 3 Results and Discussion

SI Section IV contains information on summary statistics for each of the sampling programs and each data grouping described in Table 1.

### 3.1 System-Wide Lead Concentration Assessment

**Figure 2:**
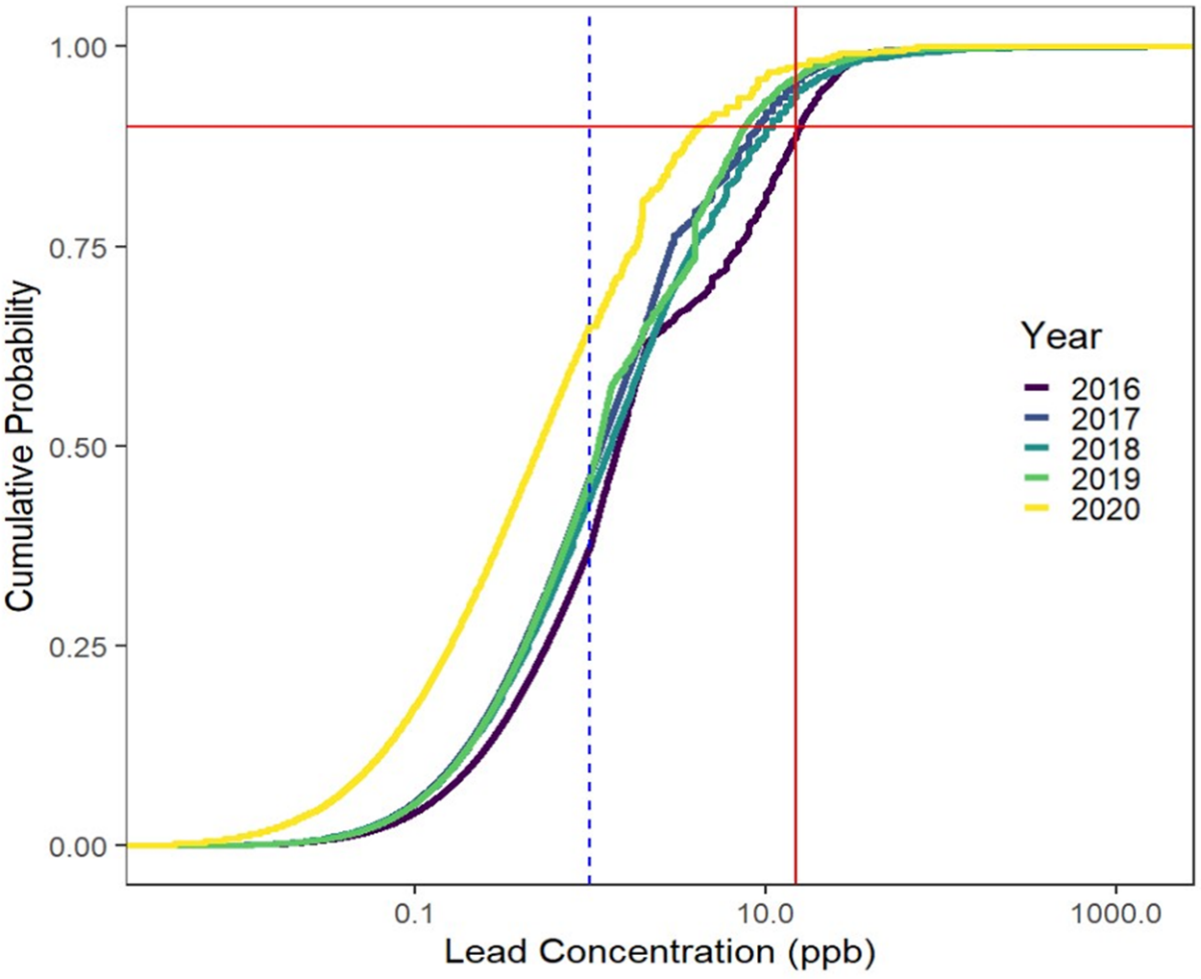
Cumulative distribution (CDF) plot of customer-requested lead sampling data by year on a logarithmic scale. The solid red vertical line represents the EPA AL of 15 ppb. The dashed blue vertical line indicates the reporting limit of 1 ppb for most of the customer-requested data. The intersection of each CDF and the solid horizontal red line indicates the corresponding 90th percentile concentration. The 90th percentiles of the distributions of customer-requested data were 15.8 ppb in 2016, 9.00 ppb in 2017, 11.0 ppb in 2018, 7.6 ppb in 2019, and 4.16 ppb in 2020.

Figure 2 shows annual customer-requested data for 2016 through 2020, plotted on a logarithmic scale. Most homes that requested samples did not have elevated levels of lead in their drinking water; the majority of the samples tested each year were below detection (56% in 2016, 73% in 2017, 76% in 2018, 54% in 2019, and 65% in 2020), even during the noncompliant periods and prior to the change to orthophosphate for corrosion control.

The customer-requested lead concentration data represent lead concentrations at specific locations in the distribution system; these locations were not selected due to any expectation that they would have lead service lines or lead in the indoor plumbing. The homes sampled each year were not selected to be representative, and the distributions of lead concentrations reported for customer-requested samples each year are statistically significantly different from all other years (see Tables S13 and S14). For example, in 2016, the 90th percentile of the customer-requested data (15.8 ppb) was above the EPA action level of 15 ppb, while in 2019 the 90th percentile lead concentration for the customer-requested distribution (7.60 ppb) was significantly below the EPA action level of 15 ppb. These differences may represent differences in the types of homes that requested sampling during different time periods. For example, homes with known lead service lines may have been early requesters of sampling (in 2016–2018), while those requesting samples in 2019 may represent homes that were less likely to be concerned (e.g., if they knew they did not have lead service lines). These differences could also represent changes in corrosion control efficacy not tied to treatment technology changes, including seasonal effects (which are explored briefly in Section III.A of the Supporting Information).

### 3.2 Corrosion Control Assessment

**Figure 3:**
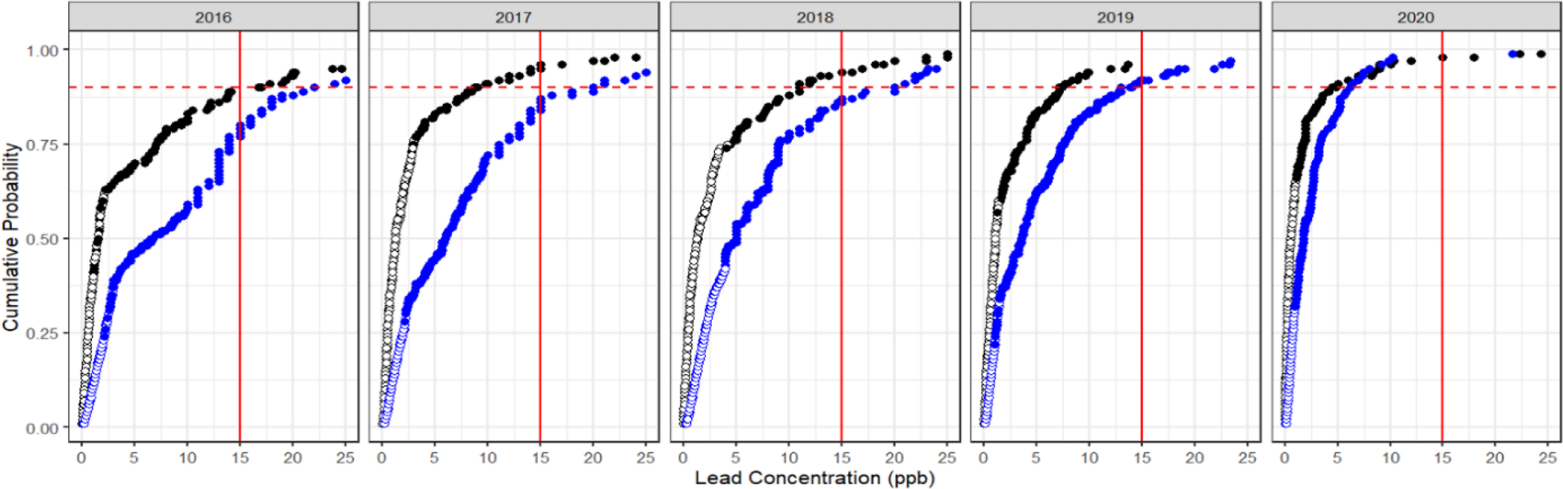
Cumulative distribution plots of the customer-requested data (black) and LCR data (blue) on an arithmetic concentration scale by year. The solid red vertical line indicates the EPA action level while the dashed red horizontal line indicates the 90th percentile concentration. The open dots within the CDFs indicate values recorded below the detection limits and have been imputed. See SI Section II for more information regarding the detection limits.

Figure 3 shows cumulative density plots of the customer-requested and LCR data for each year (Figures S31 and S33 show alternative representations). The dashed horizontal red line meets the CDF at the 90th percentile. The 90th percentile concentrations for the LCR data were above the AL (shown as a vertical red line) from 2016 to 2018; specifically, the 90th percentiles of the distributions of LCR data were 19.0 ppb in 2016, 17.0 ppb in 2017, 15.4 ppb in 2018, 12.8 ppb in 2019, and 5.14 ppb in 2020, while the customer-requested data show 90th percentiles below the AL for all years except 2016. Median values for the customer-requested samples were below laboratory reporting limits in all years (open black circles in the CDFs in Figure 3 show all imputed values), and thus, computed mean values are influenced by the imputation of data below the detection limit. For the LCR sampling, the locations were selected with the expectation that they represented homes with lead service lines or indoor lead plumbing. Nevertheless, 22% to 40% of the LCR samples taken between June 2016 and June 2019 were below the reporting limit for lead (below 2 ppb from 2016 to 2018, below 1 ppb from 2019 to 2020, and below 4 ppb for select samples in 2018) as shown by the open blue circles in the CDFs. After the introduction of orthophosphate for corrosion control, for 2020, approximately 40% of the LCR samples were below the reporting limit of 1 ppb.

Significant differences are observed in the distributions of lead concentrations between the LCR and the customer-requested data sets for all data (p < 0.001) and by corresponding year (p < 0.001) (see Table S15), with the LCR data always higher. The consistently lower values for customer-requested data compared with LCR data are different from results reported by Masten (2019) for the residential sampling conducted in Flint, MI.^9,11^ In that study, residential sampling in self-selected homes yielded higher lead concentrations than the Tier 1 LCR compliance sampling results. This might have been a result of improper collection practices for compliance samples (e.g., flushing rather than stagnant presampling) and the collection of samples at locations that were not Tier 1.^9,22−24^ Another reason that the Pittsburgh results are not similar to Flint is that Pittsburgh, PA, experienced suboptimal corrosion control from 2016 to 2019, while corrosion control chemicals were not applied in Flint, MI, from 2014 to 2015.^25,26^

Figure 3 also demonstrates significant sample censoring for water lead data. In both customer-requested and LCR sampling, many samples are below the detection limit as shown by the number of open circles. Throughout the collection period, the LCR data CDFs have a higher percentage of filled in circles than the customer-requested CDFs, showing that a higher percentage of samples in the LCR data were above the reporting limit. This is by design, with LCR samples required to be taken at Tier I sites where higher lead concentrations are expected when corrosion control is not optimized. For the customer-requested samples, the significant left-censoring (more than 1/2 of samples are represented by open circles, showing they were below detection) is due to the inclusion of all sites where a homeowner requested a sample, including many that would be expected to have very low concentrations of lead. This result explains why customer-requested data might be difficult to use for corrosion control assessment. The lead concentrations are low in some homes even when they are elevated in others, and many samples are below detection even during periods when corrosion control efficacy does not meet the LCR standard. The required LCR sampling targets Tier 1 homes because the EPA recognized that the majority of historic lead concentration data were left censored, with significant fractions of samples reported as below the detection limit.^8^ By targeting homes with lead plumbing, LCR testing focuses on those homes with the highest likelihood of experiencing elevated lead concentrations if corrosion control was not effective in the system. After the introduction of orthophosphate in 2019, the CDF of the LCR data in 2020 is shifted further to the left than in previous years, signaling a return to compliance, with many fewer samples exceeding the AL. Since the distributions of the customer-requested data for each year were always further to the left, it is not as obvious to discern this shift in lead concentrations.

The use of the 90th percentile statistic is thus supported since it is expected that Tier 1 homes sampled for LCR compliance in a system with poor corrosion control and lead service lines or plumbing will exhibit similar long-right tails. By using the 90th percentile lead concentration as the regulatory statistic to determine compliance, rather than the mean or median, the determination of compliance does not require assumptions regarding values below the detection limit.^8^ Even when a system has poorly controlled corrosion, other statistics like the mean or median may still fall below detection.

### 3.3 Effects of Orthophosphate on System-Wide Lead Concentrations

Consistent with previous studies on orthophosphate for chemical corrosion control,^21,27^ lead concentrations in the PWSA system decreased after the introduction of orthophosphate (April 2019). Customer-requested data before and after the addition of orthophosphate (samples analyzed before April 2019 and after May 2019, respectively) are significantly different (p < 0.001), and data from after the deployment of orthophosphate for corrosion control show lower lead concentrations (90th percentile values below 10 ppb in both years). Overall, the customer-requested samples are 67% below detection and have a 90th percentile value of 10.7 ppb after the introduction of orthophosphate.

Statistical tests indicate that single distributions do not appear to represent the customer-requested data adequately. This may be due to significant differences in the homes being sampled. For example, some homes may have LSLs or indoor lead plumbing or fixtures (similar to Tier I sites sampled for the LCR), while other homes may have nonlead service lines and little or no indoor lead in plumbing. While data from before orthophosphate addition are adequately represented by a single log-normal distribution (see Figure S21), data from after the change in corrosion control treatment show a poor fit to a single log-normal distribution (see Figure S22).

**Figure 4:**
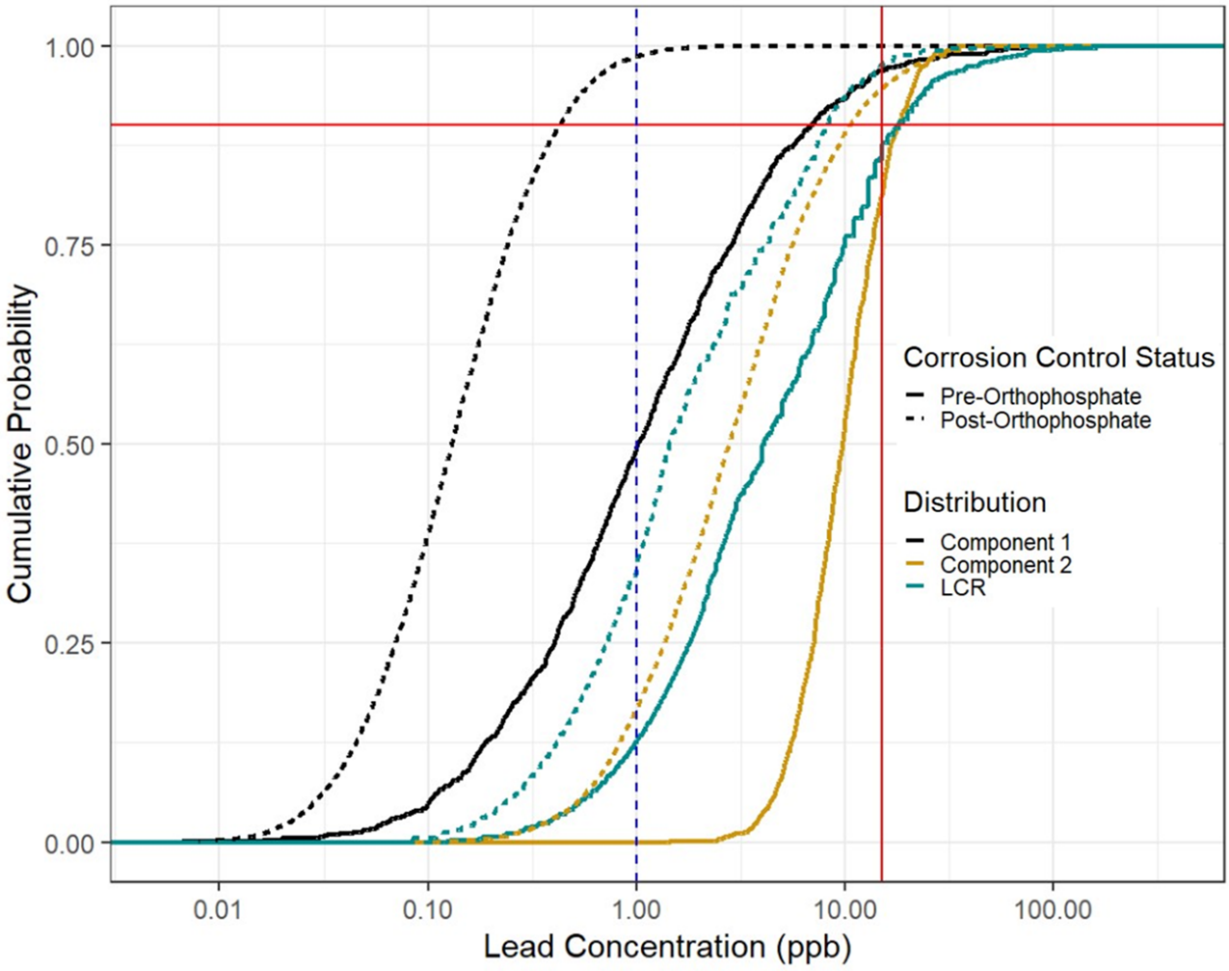
Cumulative distribution plot of lead concentrations from customer-requested and LCR data before and after the introduction of orthophosphate on a log scale. The customer-requested data are broken up into their mixture model component distributions. The solid lines indicate the pre-orthophosphate distributions, and the dashed lines indicate the corresponding post-orthophosphate distributions. The solid vertical red line indicates the EPA AL of 15 ppb. The intersection of each CDF and the solid horizontal red line indicates the corresponding 90th percentile concentration. The dashed blue vertical line indicates the lowest reporting limit of 1 ppb. SI Section II provides additional information on reporting limits.

Mixture modeling analysis indicates that two distributions represent the observed data for after the corrosion control change better than a single distribution (see Figure S24 and Tables S17 and S18). Figure 4 shows lead concentrations from LCR and customer-requested data as cumulative distribution functions before orthophosphate introduction (solid lines) and after (dashed lines) with concentrations plotted on a logarithmic scale. The customer-requested data are represented by the component distributions of the mixture model (see Figure S34 and SI Section IV.C), with black representing component 1 and yellow representing component 2, while compliance data are represented by single distributions (teal). Figure 4 indicates that before adjustment of corrosion control, the LCR data (solid teal line) fall between the two component distributions that represent the customer-requested data (solid black and solid yellow lines). Although component 2 (yellow) represents only 5% of the homes (see Table S18 and Figure S23), the distribution captures a similar 90th percentile value to the LCR distribution (17.9 ppb for component 2 and 18 ppb for the LCR distribution). Thus, the homes represented by the component 2 distribution before the application of orthophosphate may have similar characteristics as Tier 1 homes (i.e., lead plumbing or lead service lines). Prior to the corrosion control adjustment, component 1, representing 95% of the homes, was the lowest distribution of lead concentrations with a 90th percentile concentration of 6.47 ppb; 49% of the data represented by this distribution is below the detection limit (see Figure S35).

After corrosion control was changed, lead concentrations declined, with the Tier 1 samples from the LCR and the customer-requested samples showing statistically significant decreases. In Figure 4, the CDFs have all shifted left as expected. Component 1 now contains 60% of the homes and has a 90th percentile concentration below detection. Component 2 now contains 40% of the homes and has a 90th percentile of 13.1 ppb, while the LCR data after the introduction of orthophosphate have a 90th percentile concentration of 10.7 ppb. The component 2 distribution remains to the right of the LCR distribution, suggesting the lead concentrations are higher than the LCR sampled homes; the difference is statistically significant (see Table S19). Similar to the pre-orthophosphate distributions, the post-orthophosphate component 2 distribution captures homes that may have similar characteristics to the post-orthophosphate LCR distribution, suggesting that these homes may have lead service lines or plumbing like those targeted for Tier 1 sampling by the LCR. The fact that the highest customer-requested data remain above the LCR data suggests that identification of Tier 1 sampling sites is imperfect, indicating that some selected sites are not at higher risk of elevated lead concentrations when corrosion control is not optimized. This is supported by the 40% of samples below detection for the LCR sites even during the time period when corrosion control was not optimized and the system was not in compliance.

This analysis suggests that customer-requested data could be divided into two types (based on statistical analysis or on information about lead service lines), and data from this type of sampling could be used to assess corrosion compliance between regular LCR sampling times. These results also suggest that customer-requested data could be used to prioritize regions or homes for identification of service line material, service line removal, or deployment of in-home filtration (to address lead in indoor plumbing), which was attempted in Flint, Michigan, using the residential sampling data.^28^ Since the customer-requested data in this analysis did not provide exact home locations and over 33% of public side lead service line material is identified as “unknown”, a similar assessment would require significant additional analyses and information. After the successful implementation of orthophosphate in the PWSA system, most homes had very low lead concentrations (57% were below detection, and 83% were below 5 ppb). Additional analyses of the homes where lead concentrations remained above 5 ppb could highlight areas of concern in the distribution system that warrant further action.

### 3.4 Monte Carlo Simulations of Customer-Requested Data

Customer-requested data mixture models were simulated to assess how different drinking water sampling programs would affect assessment of corrosion control.

**Figure 5:**
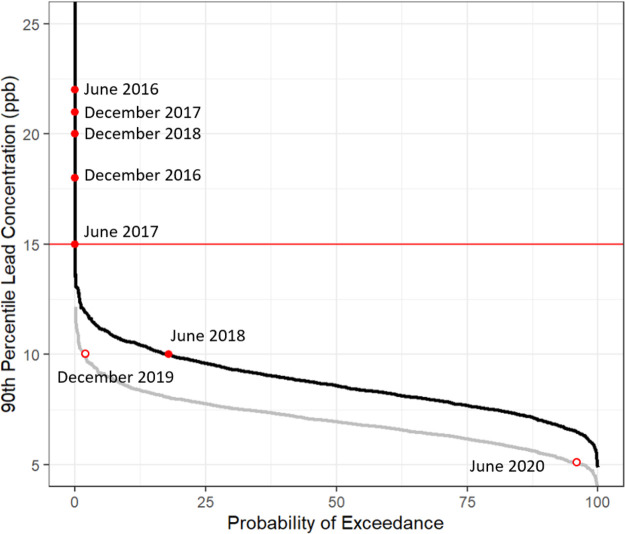
Probability of exceedance plots of the calculated 90th percentile concentrations from customer-requested Monte Carlo sampling pre- and post-orthophosphate. The black line indicates the simulated 90th percentile concentrations from the pre-orthophosphate customer-requested data, and the gray line is the post-orthophosphate customer-requested data. The solid red horizontal line indicates the EPA action level of 15 ppb. The labeled red filled dots indicate the corresponding LCR reported 90th percentile concentrations pre-orthophosphate, and the labeled red open dots indicate the 90th percentiles reported post-orthophosphate.

Figure 5 shows probability of exceedance plots of the calculated 90th percentile concentrations from the Monte Carlo simulations for the customer-requested lead sampling data pre- and post-orthophosphate. The black (pre-orthophosphate) and gray (post-orthophosphate) lines represent distributions of the calculated 90th percentiles for the simulations; these are not distributions of the raw data. The results indicate that the probability that a random sampling of 150 homes from the customer-requested data would have a 90th percentile that exceeds the AL of 15 ppb is very low (less than 1%), regardless of the status of the system. The red dots along the exceedance curves are the reported 90th percentile concentration from the LCR data, which exceed 15 ppb for all periods except June 2018. Thus, even though the system was out of compliance, determining the 90th percentile value from a randomly selected set of homes would not indicate a problem. This result was expected since most homes are not similar to Tier I homes. Even though component 2 of the pre-orthophosphate customer-requested mixture distribution was similar to the corresponding LCR distribution, it only constituted 5% of the homes sampled.

Post-orthophosphate the reported 90th percentiles from the LCR data fall within the lower and upper tail of the exceedance curve of simulated 90th percentiles from the customer-requested data. The LCR 90th percentile reported in December 2019 of 10 ppb is *greater than* 98% of the simulated customer-requested 90th percentiles, while the 5.1 ppb reported by the utility in June 2020 is *less than* 96% of the simulations (>4%). Optimization of orthophosphate corrosion control typically takes over a year, and as a result, lead concentrations tend to drop as dosing of orthophosphate is tuned to optimal levels.^21^ Since the reported LCR 90th percentile values were sampled at two distinct periods, and the customer-requested data were collected continuously after orthophosphate was introduced, it is not surprising that the 90th percentile reported in June 2020 is much lower than the simulated 90th percentiles based on customer-requested data from June 2019 through June 2020, which capture data from when the system was adjusting to the addition of orthophosphate. This is shown in Figure S36 which provides simulated customer-requested 90th percentile concentrations by year.

These results indicate that the 90th percentile concentrations simulated from customer-requested sampling prior to corrosion control optimization rarely capture the 90th percentile concentration reported from the targeted Tier 1 sampling conducted for LCR compliance. Since the system sampled in the current study was undergoing corrosion control optimization, it is difficult to make conclusions on whether the post-orthophosphate simulations captured the 90th percentile concentrations reported by the utility. Follow-up LCR and customer-requested sampling after corrosion control is optimized may elucidate if simulated customer-requested 90th percentile concentrations resemble reported LCR 90th concentrations once optimal corrosion control has been achieved for a longer duration.

This analysis indicates that while there are homes that resemble the Tier 1 homes from LCR sampling, in general, customer-requested sampling programs are insufficient to assess corrosion control efficacy. Tier 1 residential sampling results in greater 90th percentile concentrations than random customer-requested sampling does, especially when the utility is out of compliance, and thus, targeted Tier 1 sampling remains the best method to assess corrosion control. However, customer-requested sampling remains a useful way to identify homes to target for lead service line replacement and further remediation efforts, especially if concentrations of lead at a home remain elevated after corrosion control has been optimized.

### 3.5 Spatial Sampling Bias Assessment of Customer-Requested Data

The prior analyses looked at how different sampling programs captured temporal variability in lead concentrations resulting from adjustments to corrosion control. Geographic variability of drinking water lead concentrations has also been studied to understand the causes of elevated lead levels. The age of homes, presence of lead plumbing and service lines, and socio-economic indicators have all been statistically significantly linked to elevated lead levels in drinking water for cities with recent LCR exceedances.^6,7,28,29^ These factors can also lead to nonrandom results in a self-selected sampling program, like the customer-requested sampling program.^11^

**Figure 6:**
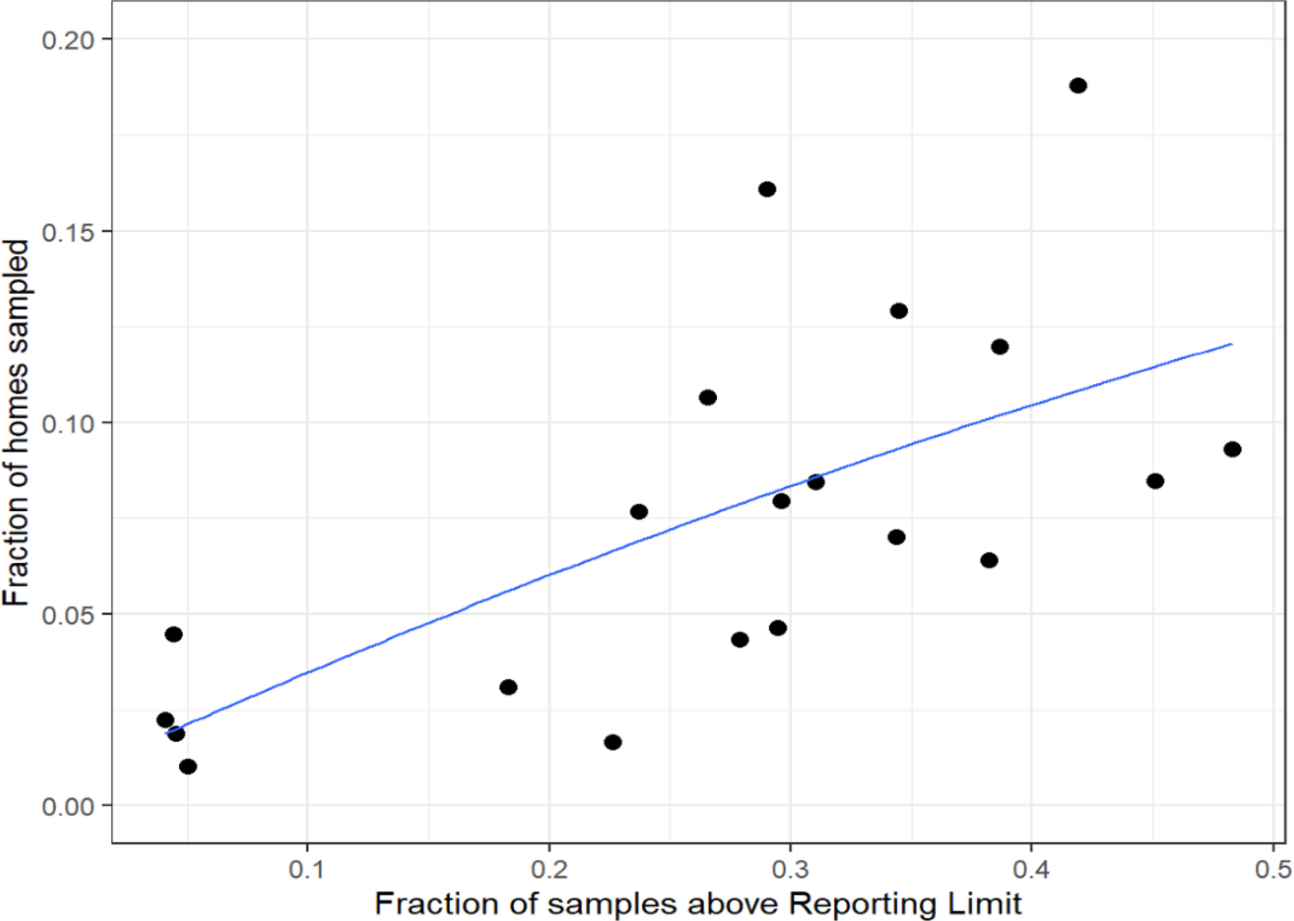
Scatterplot of the fraction of samples above the reporting limit and the fraction of homes sampled within a ZIP code. Each dot signifies a ZIP code within the distribution network, and the blue quadratic line fit to the data represents the general increasing trend of homes sampled as sample results return above the laboratory reporting limit.

Geographic variability was observed in both the fraction of customer-requested samples above the detection limit and the fraction of homes sampled per ZIP code as shown in Figure 6. Each dot represents a ZIP code within PWSA’s distribution network. The results indicate that as the fraction of samples testing above the detection limit in a ZIP code increases, the fraction of homes that are sampled within that ZIP code increases as indicated by the blue quadratic line fit to the data. This increased self-selection for sampling within specific ZIP codes may be the result of individuals hearing about neighbors having high lead concentrations, public information campaigns about the prevalence of lead service lines or plumbing within the area, or the prevalence of children in the area. Further analysis of spatial covariates is warranted, and the intersection of temporal variability and spatial variability should be assessed.

## Supporting Information Available

Additional description of study city; additional descriptive statistics and model fit analyses, tables and figures; results of the statistical tests of difference; additional Monte Carlo sampling analyses by year; and additional maps of the spatial bias assessment (PDF)
