# Supplementary material for: Assessment of Lead in Drinking Water from Multiple Drinking Water Sampling Programs for a Midsize City
Source: Environ Sci Technol. 2022 Dec 23;57(1):842–51. doi: 10.1021/acs.est.2c06614 (PMC9835881; doi:10.1021/acs.est.2c06614)
Supplement: Supplementary file 1 — es2c06614_si_001.pdf [file es2c06614_si_001.pdf]

Supplemental Information

Title: **Assessment of lead in drinking water from multiple drinking water sampling programs for a mid-size city**

Authors: *Vasikan Vijayashanthar*†; Mitchell J. Small*†, ‡; *Jeanne M. VanBriesen*†, ‡

† Department of Civil and Environmental Engineering, Carnegie Mellon University, 5000 Forbes Avenue, Pittsburgh, Pennsylvania, 15213, United States

‡ Department of Engineering and Public Policy, Carnegie Mellon University, 5000 Forbes Avenue, Pittsburgh, Pennsylvania, 15213, United States

*Denotes corresponding author. Contact email: vvijayas@andrew.cmu.edu.

ORCID

Vasikan Vijayashanthar: 0000-0002-5736-4580

Jeanne VanBriesen: 0000-0002-2631-0213

68 Pages

23 Tables

38 Figures

Table of Contents

I. Background 3

A. LCR Requirements 3

B. Study Area 3

II. Imputation of below reporting limit values 6

III. Statistical Analyses 12

A. LCR Compliance Sampling Data 12

B. Customer-Requested (Household) Lead Sampling Data 22

C. System-Wide Lead Concentration Assessment: Customer-requested Lead Concentrations 33

D. System-Wide Corrosion Control Assessment: Household and LCR Compliance Concentrations 34

E. Pre- and Post-Orthophosphate Data 36

F. Monte Carlo Simulations 45

G. Spatial customer-requested bias assessment 47

IV. Results and Discussion 48

A. System Wide Lead Concentrations: Customer Requested Concentration Data 48

B. System Wide Corrosion Control Assessment: Customer Requested and LCR Compliance Data 53

C. Pre- and Post-Orthophosphate 61

D. Monte Carlo simulations of the customer-requested data 64

E. Spatial sampling bias assessment of customer-requested data 66

I. Background

A. *LCR Requirements*

The LCR provides requirements and guidelines for public water systems (PWS) to assess and control the corrosion of lead in drinking water systems. The LCR required public water systems serving more than 50,000 people to install corrosion control treatment (CCT) by January 1, 1997, with limited exceptions. PWSs serving less than 50,000 people that exceeded the lead and/or copper action limit were required to install CCT. CCT options included alkalinity and pH adjustment, calcium hardness adjustment, and phosphate or silicate-based corrosion inhibitors.

Sampling requirements to monitor lead concentrations in the PWS and determine the effectiveness of CCT varies based on the number of people served by the utility (i.e., 100 samples for a system serving > 100,000 people). At the time of promulgation, systems were required to collect samples semi-annually. Systems could qualify for annual or triennial monitoring at a reduced number of sites based on the number of consecutive years meeting the LCR requirements. Systems serving less than 3,300 homes could apply for a 9-year monitoring waiver.[1]

B. *Study Area*

Pittsburgh is located at the confluence of the Allegheny and Monongahela rivers, which form the Ohio river. The Pittsburgh Water and Sewer Authority (PWSA) provides water to approximately 300,000 customers throughout the City of Pittsburgh, producing on average 265 million liters (70 million gallons) of water per day. The system contains approximately 1,550 kilometers (965 miles) of water lines, five reservoirs and 11 tanks amounting to a water storage capacity of 1,720 million liters (455 million gallons). Much of the distribution system was built

around the 1920’s. The shaded green area in **Figure S1** is the boundary of the water distribution network that the utility serves within the Pittsburgh city limits.

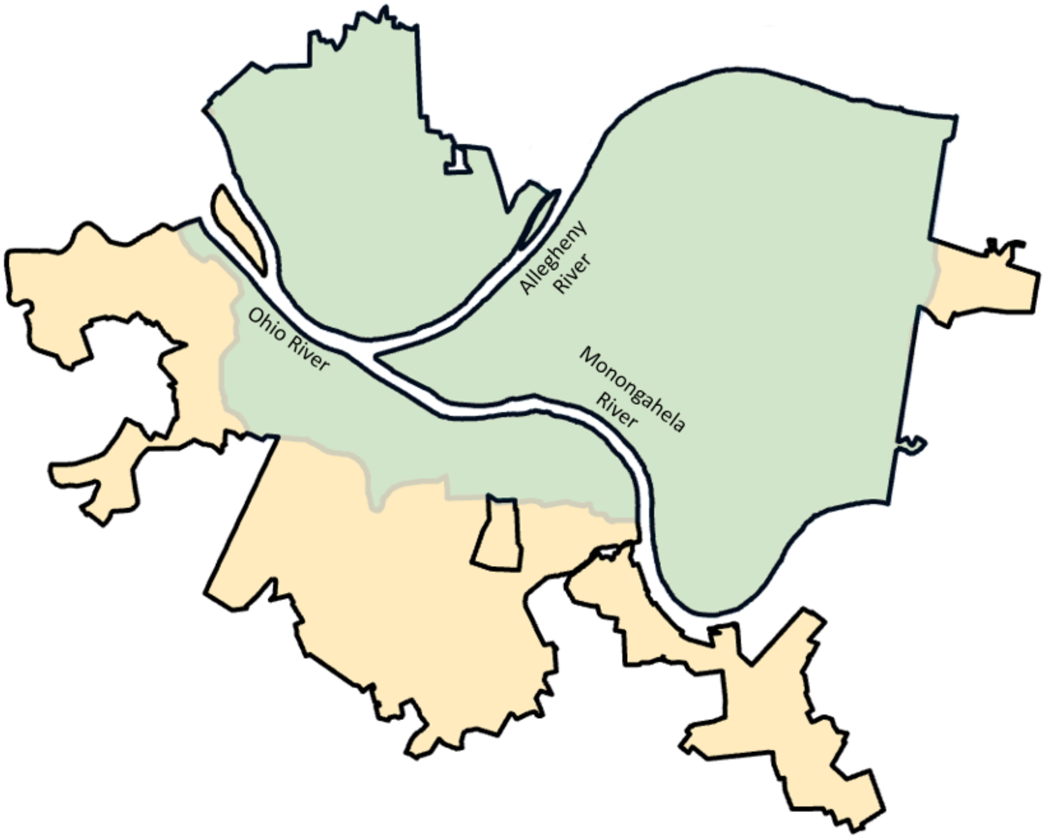

Figure S1: Map of Pittsburgh city limits with the utility's water service area shaded in green. The three rivers are labeled.

The present study focuses on PWSA because the utility reported an exceedance of the Action Level (90th percentile >15ppb) in June 2016 during routine triennial LCR sampling. PWSA had been using soda ash for corrosion control prior to the exceedance and continued using it until approval from the state to switch to orthophosphate.[2] There was a change to caustic soda (sodium hydroxide) for pH control in April 2014 that was reversed by the utility in January 2016.[3] Lead concentrations measured at homes from 2016 through 2020 thus represent a time period when the utility was working to improve corrosion control and take other corrective measures required by the LCR. The utility began a lead service line inventory and a lead service

line replacement (LSLR) program, and between June 2016 and July 2020, the utility replaced over 7,400 public lead service lines and more than 4,700 private lead service lines.[4]

II. Imputation of below reporting limit values

Semi-parametric lognormal regression on order statistics (ROS) was used to account for samples below the reporting limit in all data sets. The LCR data measurements were based on laboratory analysis with a reporting limit of 2 ppb from June 2016 to June 2018 and 1 ppb from December 2018 to June 2020, except for 37 of the 161 samples in the December 2018 reporting period, which had a reporting limit of 4 ppb (23 percent of samples in this reporting period). This most likely indicates that multiple laboratories with varying reporting limits were used during these compliance periods. All samples below the detection limit were reported by the utility as 0 ppb. ROS was performed for each compliance period of the LCR data using the corresponding reporting limit, as shown in **Table S1**. Values recorded as 4 ppb in the December 2018 reporting period were imputed as well, but all values recorded *between* 1 ppb and 4 ppb were assumed to be detected at the reported concentrations and were not imputed. **Table S1** provides the number of below reporting limit samples, and in parentheses, the corresponding fraction of the samples that were below detection for the LCR Compliance data.

The determination of reporting limits for the customer-requested data was more complicated. Multiple laboratories were used from 2017 to 2019 to meet demand for the customer-requested water testing program. Reporting limits for each sample were not provided, and below detection limit samples were not always identified by reporting zero. Thus, for each customer-requested sample reported, the clustering of lead concentrations by year was used to infer the relevant reporting limit for the laboratories utilized that year. These raw data include some samples that were reported in the customer-requested data set as 0 ppb; these samples were assumed to represent below detection limit results. **Table S2** provides the reporting limits used by the laboratories for each year of household sample collection. Previous studies using earlier

versions of this dataset identified reporting limits that were largely consistent with the reporting limits identified in Table S2.[5]

Table S3 provides the frequency of the suspected reporting limit lead concentrations by year, and in parentheses, the corresponding fraction of the total sample size. A semi-parametric lognormal regression on order statistics method was used to impute values below the lowest corresponding reporting limit by year and at each subsequent reporting limit for the customer requested data. Values that were recorded between reporting limits were assumed to be detected concentrations and were not imputed since some laboratories were capable of measuring such concentrations.

It is important to note that there are multiple ways to deal with values below reporting or detection limits. The ROS imputation method was compared to replacement techniques which include replacement of values with ½ the reporting limit, the reporting limit itself, or even zero. The implications of these replacement techniques and the ROS method are presented in **Table S4** and **Table S5**. The table indicates that the percent difference in means from the ROS to replacement method using the largest reporting limit is between 6 and 60% for the 5 years of customer-requested sampling data. The LCR data exhibit much smaller percent difference for the 5 years of data between 1 and 14%. When considering the median, the LCR compliance data exhibited no change when using replacement techniques and the ROS imputation, this is because for all but one reporting period more than half of the data was above the detection limit. The customer-requested data on the other hand did exhibit differences ranging from 13 to 260% in the medians between the two techniques. It is important to note that much of the analysis included in the paper draws conclusions about the data above the detection limit and using the

90th percentile statistic. These results would not be affected by the use of replacement or imputation techniques.

Table S1: Number of samples and frequency of samples reported below reporting limits for each reporting period of LCR Compliance data.

	Reporting Period								
Reporting Limit(ppb)	June 2016	Dec. 2016	June 2017	Dec. 2017	June 2018	Dec. 2018	June 2019	Dec. 2019	June 2020
1	0 (0.00)	0 (0.00)	0 (0.00)	0 (0.00)	0 (0.00)	**31 (0.19)**	**39 (0.22)**	**65 (0.39)**	**63 (0.40)**
2	**45 (0.45)**	**52 (0.33)**	**46 (0.36)**	**38 (0.32)**	**57 (0.54)**	0 (0.00)	0 (0.00)	0 (0.00)	0 (0.00)
4	0 (0.00)	0 (0.00)	0 (0.00)	0 (0.00)	0 (0.00)	**37 (0.23)**	0 (0.00)	0 (0.00)	0 (0.00)

Table S2: Reporting limits for each year of Customer Requested Lead (household) sampling data collection (provided with the data).[6]

**Year**	**Reporting Limits (ppb)**	**Count of samples below Reporting Limit (n)**
2016	1	1623 (2901)
2017	1, 2	4617 (6327)
2018	1, 2, 4	2309 (1753)
2019	1, 4	1294 (2410)
2020	1	225 (347)

Table S3: Number of samples and frequency of samples reported at assumed reporting limits for each year of customer requested lead sampling data

	Year				
Lead Concentration of Reporting Limits (ppb)	2016	2017	2018	2019	2020
0	**1621 (0.61)**	**2029 (0.30)**	**11 (0.00)**	0 (0.00)	0 (0.00)
1	2 (0.00)	92 (0.01)	72 (0.03)	**1290 (0.57)**	**225 (0.65)**
2	6 (0.00)	**2487 (0.36)**	**865 (0.38)**	20 (0.01)	7 (0.02)
4	3(0.00)	54 (0.01)	**653 (0.29)**	**105 (0.05)**	1 (0.00)

Table S4:Comparison of means from replacement and ROS of data below reporting limits for the LCR and customer-requested data by year

		**Using ROS imputation**	**Using 0**	**Half of the (largest) reporting limit**	**Largest reporting limit**	**Percent Difference between using ½ and ROS**	**Percent Difference between largest and ROS**

Year	Sample Set	Mean					
2016	Customer-Requested	5.41	4.96	6.08	7.21	12.4	33.3
	LCR Compliance	9.33	8.77	9.14	9.51	2.06	1.91
2017	Customer-Requested	3.79	3.13	4.60	6.07	21.4	60.2
	LCR Compliance	9.96	9.58	9.92	10.3	0.43	3.00
2018	Customer-Requested	5.54	4.95	6.34	7.73	14.4	39.5
	LCR Compliance	7.16	6.11	7.13	8.16	0.37	14.0
2019	Customer-Requested	3.77	3.48	3.75	4.02	0.53	6.63
	LCR Compliance	6.27	6.07	6.22	6.37	0.77	1.64
2020	Customer-Requested	2.17	1.97	2.30	2.62	5.99	20.7
	LCR Compliance	2.23	2.03	2.23	2.43	0.15	8.79

Table S5: Comparison of medians from replacement and ROS of data below reporting limits for the LCR and customer-requested data by year.

		Using ROS imputation	Using 0	Half the (largest) reporting limit	Largest reporting limit	Percent Difference between using ½ and ROS	Percent Difference between largest and ROS
Year	Sample Set	Median					
2016	Customer-Requested	1.44	0	0.5	1	65.3	30.6
	LCR Compliance	4.3	4.3	4.3	4.3	0	0
2017	Customer-Requested	1.15	0	1	2	13.0	73.9
	LCR Compliance	4.2	4.2	4.2	4.2	0	0
2018	Customer-Requested	1.31	0	2	4	52.7	205

	LCR Compliance	4	0	2	4	50.0	0
**2019**	Customer-Requested	1.11	0	2	4	80.2	260
	LCR Compliance	2.73	2.73	2.73	2.73	0	0
**2020**	Customer-Requested	0.51	0	0.5	1	1.96	96.1
	LCR Compliance	1.32	1.32	1.32	1.32	0	0

III. Statistical Analyses

A. *LCR Compliance Sampling Data*

Within each data set, statistical tests of similarity between pairs of different sampling periods (by reporting period for LCR and by year for customer requested data) were performed. For each LCR compliance period, the mean ranks of the sampled distributions were evaluated for statistically significant differences using pairwise Wilcoxon rank sum tests, shown in **Table S6**. An alternative hypothesis that the two mean ranks were unequal was used.[7] For comparisons between the distributions of lead concentrations for each compliance period, **Table S7** provides the results of pairwise Kolmogorov-Smirnov tests for statistically significant differences in the distributions.[8] An alpha value of 0.05 was used to determine statistical significance.

The Wilcoxon rank sum test results indicate that both the preceding compliance period and the succeeding compliance period are statistically significantly different from each compliance period assessed, starting in 2018. In Table S4, the furthest left diagonal values beginning in 2018 are all below 0.05, indicating there is enough evidence to reject the null hypothesis, supporting the alternative hypothesis that the sample mean ranks are statistically significantly different. This result suggests there are statistically significant differences in lead concentration distributions in compliance samples reported from 2018 onward. Also, from 2016 to 2018, lead concentrations reported in December have statistically significantly higher means and medians than those reported in June of the same year. This may be the result of sampling in warmer months like July-September, during which higher lead concentrations are expected.[9] Statistically significant seasonal differences were not observed in 2016 to 2017. Differences observed after the June 2019 reporting period may be influenced by the introduction of orthophosphate corrosion control, which is suggested by the median and mean of the reported

concentration distribution in December 2019 being lower than in June 2019. Since samples collected in 2016 to 2017 do not exhibit seasonality, and samples from 2019 and 2020 are most likely statistically significantly different due to the introduction of orthophosphate, seasonal effects are not clear throughout the time period of the study. Annually aggregated compliance data were used in all subsequent analyses.

Further, the lead concentration distribution reported in June 2020 is statistically significantly different from the distributions reported in all other lead compliance periods (all June 2020 values in **Table S6** are below 0.05). This corresponds to the introduction of orthophosphate in 2019 and subsequent decrease in lead concentrations observed.

The results of the pairwise Kolmogorov-Smirnov tests are very similar to the Wilcox-Signed rank test results, indicating that statistically significant differences in lead concentration distributions are observed after June 2018 as indicated by the bolded cells above and below the diagonal identity values. Since differences after 2019 may be the result of orthophosphate introduction, and statistically significant differences weren't observed in 2016 and 2017, this analysis also supports the use of annually aggregated samples for all subsequent analysis. The results also indicate that the distribution of lead concentrations reported in June 2020 is statistically significantly different from all other compliance periods, since the bottom and furthest left row of values in **Table S7** are all below 0.05; again, this is expected since the introduction of orthophosphate in 2019 was expected to reduce lead concentrations.

Table S6: Pairwise Wilcoxon Rank Sum Test statistics to test for statistically significant differences in lead concentration means for each LCR sampling period. Alpha values are provided, any value below 0.05, bolded below, indicates that there is enough evidence to reject the null hypothesis that the sample mean ranks are the same.

Reporting Period	2016 Jan - June	2016 July - Dec	2017 Jan - June	2017 July - Dec	2018 Jan - June	2018 July - Dec	2019 Jan - June	2019 July - Dec
2016 July - Dec	0.09	-	-	-	-	-	-	-
2017 Jan - June	0.72	**0.01**	-	-	-	-	-	-
2017 July - Dec	0.51	0.28	0.25	-	-	-	-	-
2018 Jan - June	0.08	**0.00**	0.08	**0.00**	-	-	-	-
2018 July - Dec	0.08	0.90	**0.02**	0.33	**0.00**	-	-	-
2019 Jan - June	0.35	**0.00**	0.40	**0.05**	0.34	**0.00**	-	-
2019 July - Dec	**0.00**	**0.00**	**0.00**	**0.00**	0.28	**0.00**	**0.04**	-
2020 Jan - June	**0.00**	**0.00**	**0.00**	**0.00**	**0.00**	**0.00**	**0.00**	**0.00**

Table S7: Associated p-values of pairwise Kolmogorov-Smirnov test for statistically significant differences in lead concentration distributions among LCR sampling periods. Alpha values below 0.05, bolded, indicate that there is enough evidence to reject the null hypothesis that the sample distributions are the same.

Reporting Period	2016 Jan - Jun	2016 July - Dec	2017 Jan - Jun	2017 July - Dec	2018 Jan - Jun	2018 July - Dec	2019 Jan - Jun	2019 July - Dec	2020 Jan - Jun
2016 Jan - Jun	1.00	0.15	0.38	0.63	**0.01**	**0.01**	0.10	**0.00**	**0.00**
2016 July - Dec	0.15	1.00	0.05	0.53	**0.00**	0.08	**0.00**	**0.00**	**0.00**
2017 Jan - Jun	0.38	0.05	1.00	0.36	**0.02**	**0.01**	0.44	**0.00**	**0.00**
2017 July - Dec	0.63	0.53	0.36	1.00	**0.00**	0.12	0.06	**0.00**	**0.00**
2018 Jan - Jun	**0.01**	**0.00**	**0.02**	**0.00**	1.00	**0.00**	0.33	0.06	**0.00**
2018 July - Dec	**0.01**	0.08	**0.01**	0.12	**0.00**	1.00	**0.00**	**0.00**	**0.00**
2019 Jan - Jun	0.10	**0.00**	0.44	0.06	0.33	**0.00**	1.00	**0.02**	**0.00**
2019 July - Dec	**0.00**	**0.00**	**0.00**	**0.00**	0.06	**0.00**	**0.02**	1.00	**0.00**
2020 Jan - Jun	**0.00**	**0.00**	**0.00**	**0.00**	**0.00**	**0.00**	**0.00**	**0.00**	1.00

Figures S2 to S6 show the normal distribution fits to the log-transformed LCR compliance data by year. Each of these plots include histograms of empirical data with the theoretical density function of the fitted normal distribution (top left), quantile-quantile plots (top right), empirical and theoretical CDFs (bottom left) and probability-probability plots (bottom right). If the fitted theoretical density follows the shape of the empirical histogram, the normal distribution is likely a good fit. Alignment of the dots with the identity line in the quantile-quantile and probability plots also indicate a good fit. If the dots deviate from the line, there is an indication that the range of the sample set is not well represented by the selected model. Alignment of the empirical cumulative distribution dots with the theoretical cumulative distribution line indicates a good fit.

Reviewing **Figures S2** to **S6**, the qualitative assessment of the LCR compliance data fits to a single lognormal distribution indicate good representations of the data when separated by year. The fitted theoretical densities are typically in good alignment with the histograms of the empirical data. Quantitative goodness of fit tests were also performed to assess if the distributions were normally distributed (again, after log-transformation), including the Lilliefors test, Akaike's Information Criteria (AIC) and Bayesian Information Criteria (BIC). [8], [10] These results (see **Table S8** below), indicate that the log-transformed annual LCR compliance data fit a normal distribution well.

Statistical tests of similarity between the different years of LCR compliance sampling data were performed using pairwise Wilcoxon rank sum tests and pairwise Kolmogorov-Smirnov tests. **Table S9** and **Table S10** provide the resulting p-values for the tests of similarity. The statistical tests indicate that annually aggregated LCR sample mean ranks are similar in 2016, 2017 and 2018 but are statistically significantly different in 2019 and 2020. A similar result is

observed in the distributions of lead concentrations per year, as only 2019 and 2020 are statistically significantly different from the other years of lead compliance distributions. As noted above, this is most likely the result of the introduction of orthophosphate and efforts to optimize corrosion control taken in April 2019.

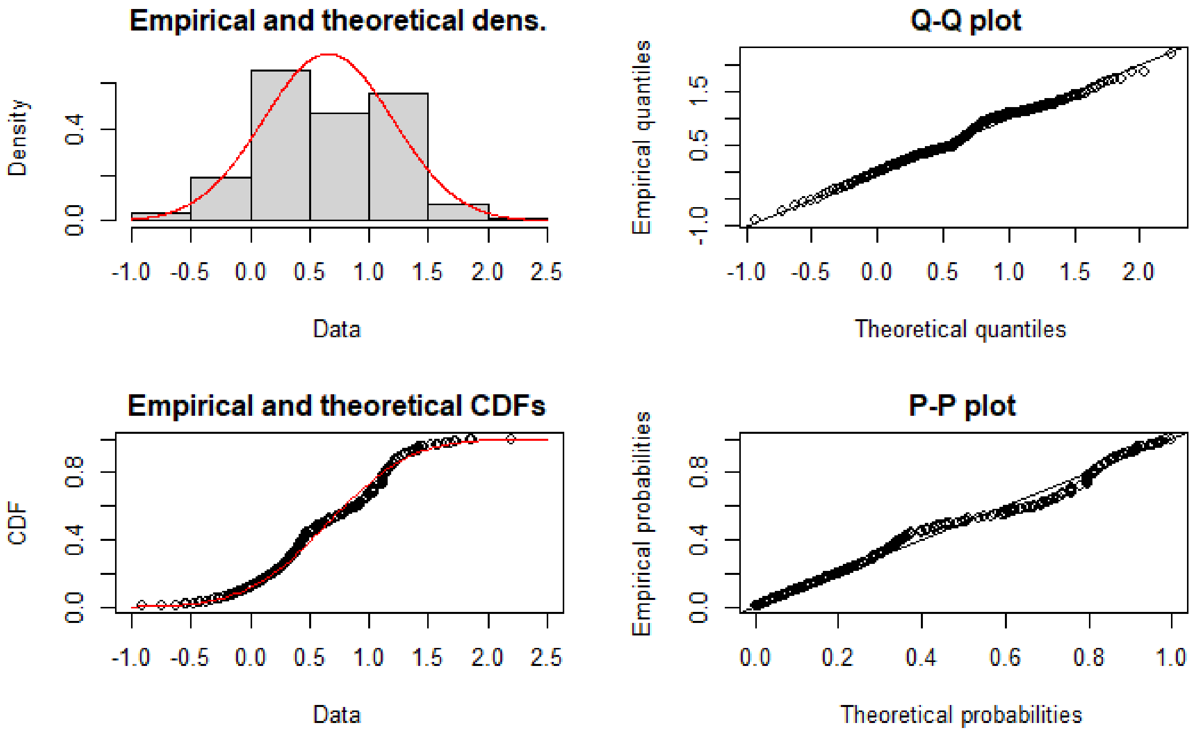

Figure S2: Goodness of fit plots for normal distribution fitting of LCR data sampled in 2016.

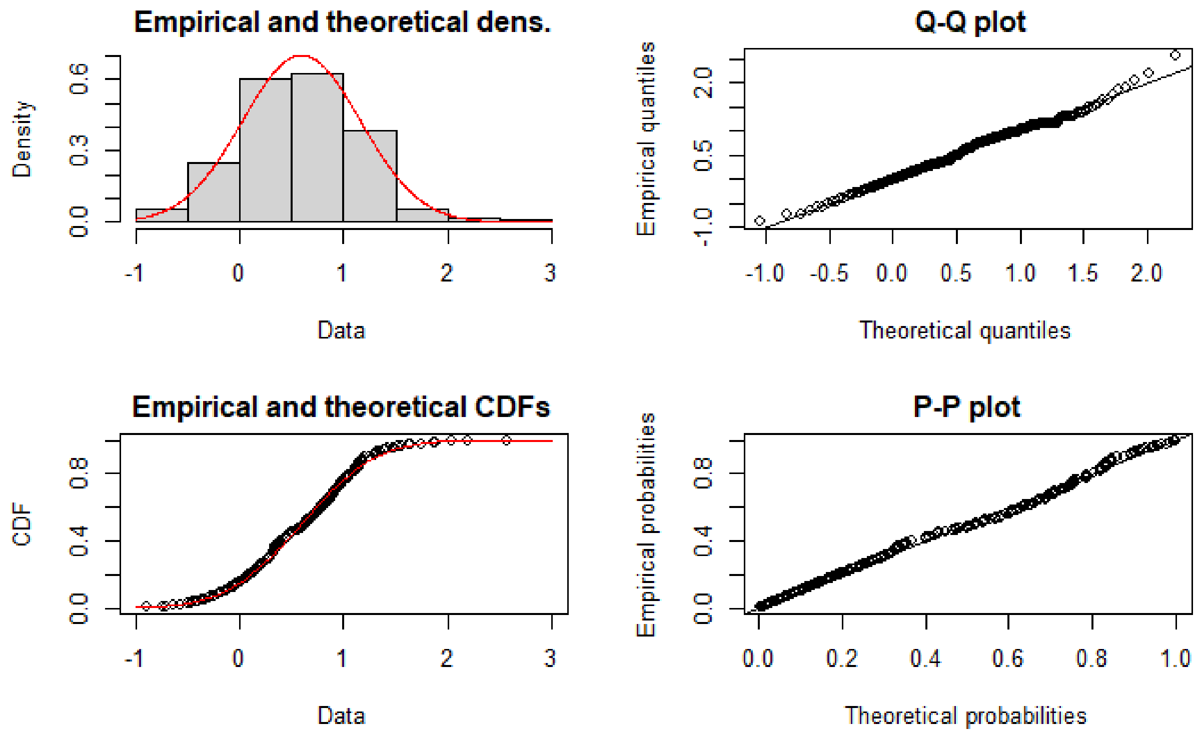

Figure S3: Goodness of fit plots for normal distribution fitting of LCR data sampled in 2017.

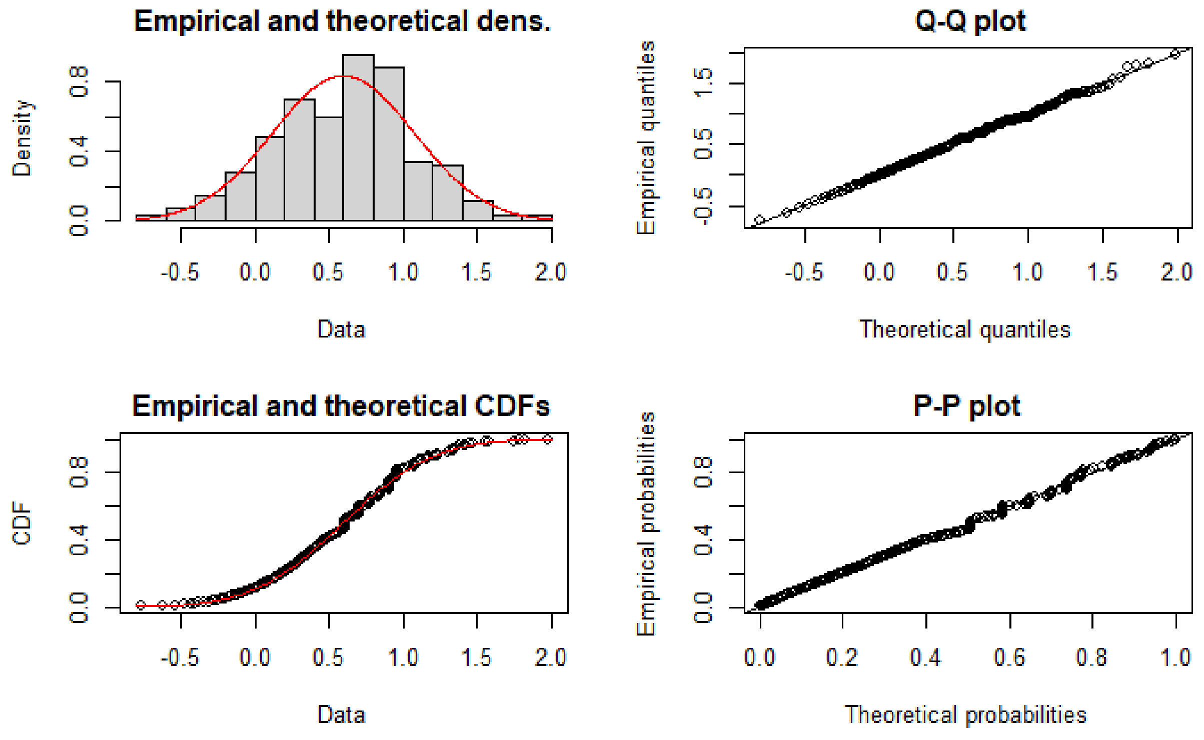

Figure S4: Goodness of fit plots for normal distribution fitting of LCR data sampled in 2018.

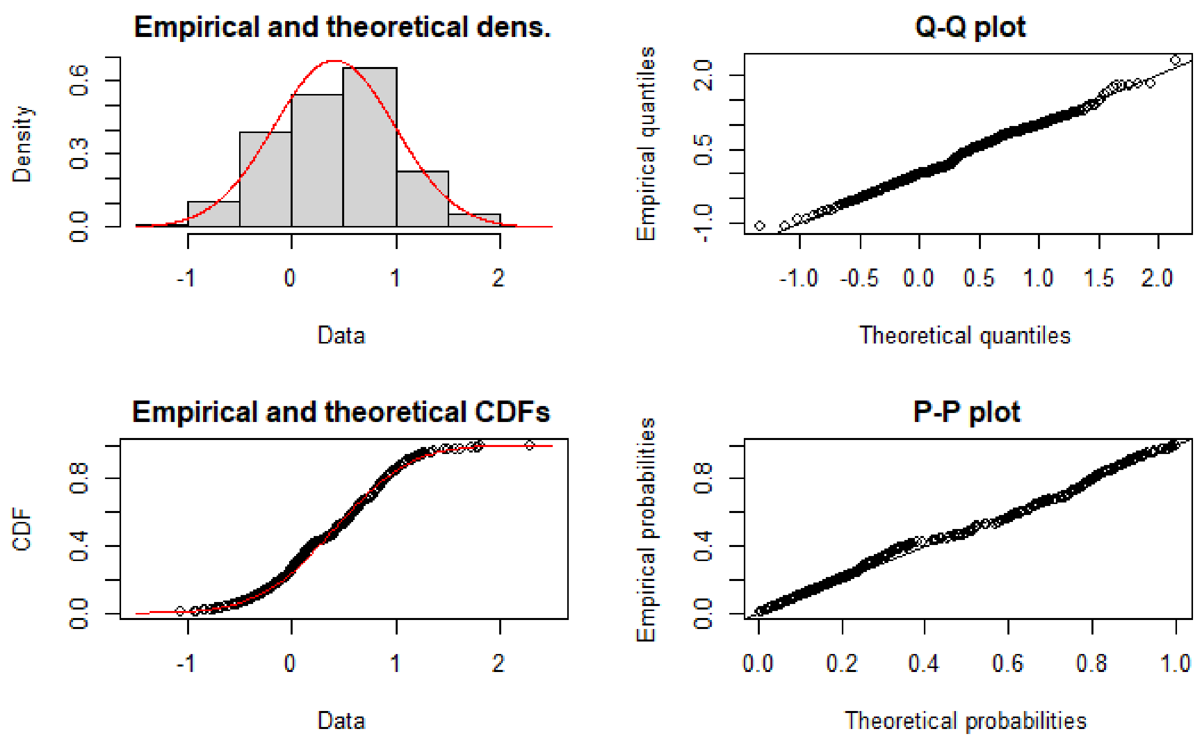

Figure S5: Goodness of fit plots for normal distribution fitting of LCR data sampled in 2019.

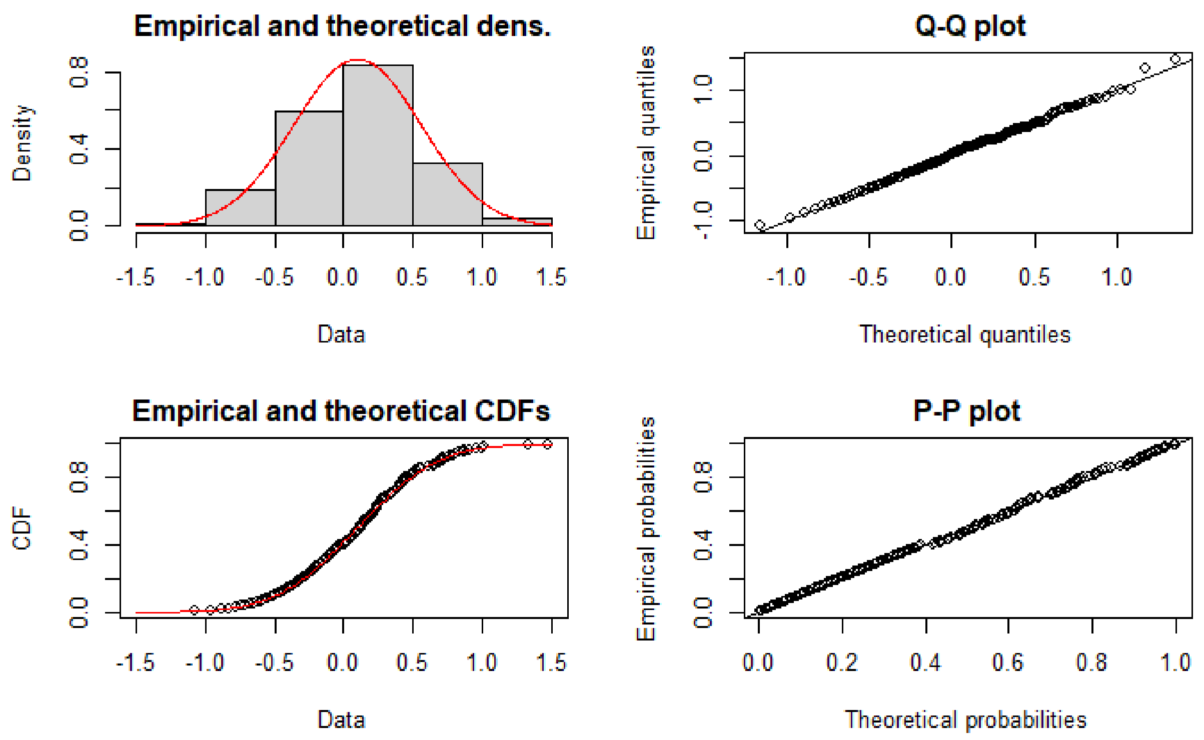

Figure S6: Goodness of fit plots for normal distribution fitting of LCR data sampled in 2020.

Table S8: Parameters and goodness of fit statistics for fitted normal distribution to log transformed LCR lead sampling data by year.

Year	Goodness of Fit Statistics				Normal Parameters	
	BIC	AIC	Lilliefors test	KS test	Mean	Standard Deviation
2016	433	426	0.08	not rejected	0.66	0.55
2017	432	425	0.05	not rejected	0.59	0.57
2018	377	369	0.04	not rejected	0.60	0.48
2019	617	609	0.05	not rejected	0.41	0.58
2020	214	208	0.03	not rejected	0.10	0.46

Table S9: Results of pairwise Kolmogorov-Smirnov test for statistically significant differences in lead concentration distributions among LCR Compliance sampling data by year. Alpha values below 0.05, bolded, indicate that there is enough evidence to reject the null hypothesis that the sample distributions are the same.

**Year**	**2016**	**2017**	**2018**	**2019**	**2020**
**2016**	1.00	0.09	**0.00**	**0.00**	**0.00**
**2017**	0.09	1.00	0.22	**0.00**	**0.00**
**2018**	**0.00**	0.22	1.00	**0.00**	**0.00**
**2019**	**0.00**	**0.00**	**0.00**	1.00	**0.00**
**2020**	**0.00**	**0.00**	**0.00**	**0.00**	1.00

Table S10: Pairwise Wilcoxon rank sum test statistics to test for statistically significant differences in lead concentration means for each year of LCR compliance sampling. Alpha values are provided; any value below 0.05, bolded, indicates that there is enough evidence to reject the null hypothesis that the sample mean ranks are the same.

**Year**	**2016**	**2017**	**2018**	**2019**
**2017**	0.15	-	-	-
**2018**	0.15	0.98	-	-
**2019**	**0.00**	**0.00**	**0.00**	-
**2020**	**0.00**	**0.00**	**0.00**	**0.00**

B. *Customer-Requested (Household) Lead Sampling Data*

For each data grouping for the customer-requested data, the data were log transformed and a normal distribution was fit. Data were grouped by year using analysis date (2016, 2017, 2018, 2019 and 2020) as well as by pre-orthophosphate introduction (Feb 2016 – March 2019) and post-orthophosphate introduction (June 2019 - June 2020). For these customer-requested data, the distributions represent the lead in water within the city during each time period, without consideration of whether the homes were likely to have lead service lines or indoor lead plumbing.

Figures S7 to S12 show the normal distribution fits to the log-transformed customer-requested data by year (2016 to 2020). Although qualitative assessment of the fitted distributions indicates a reasonable representation of the data, quantitative test results including Lilliefors test statistics and Kolmogorov-Smirnov test results provided in **Table S11** indicate a poor fit.

For the customer-requested data, in several cases, single distributions do not appear to represent the data adequately (2016, 2017, and 2019). Two-component mixture models were assessed for all cases to compare.

The mathematical form of the probability density function of a finite mixture model is provided below.[11]

$$f(x) = \sum_{i=1}^{k} \pi_i p_i(x)$$

Where π_i is the mixture weight for the probability density function of each subpopulation $p_i(x)$.

Figures S13 to S18 show the mixture model fits. Results of the quantitative statistical tests on the mixture results, including Kolmogorov-Smirnov Test and BIC, are included in **Table**

S12. For these customer-requested data, qualitative and quantitative assessment of the relative goodness of fit, the mixture model fits were an improvement over single distribution fit in every case except the full data set (all years). In some cases, this is quite clear in the visuals (e.g., 2016, **Figure S13**), while in other cases, the improvement in fit is slight and single log-normal distribution fits were adequate (e.g., 2020, **Figure S17**).

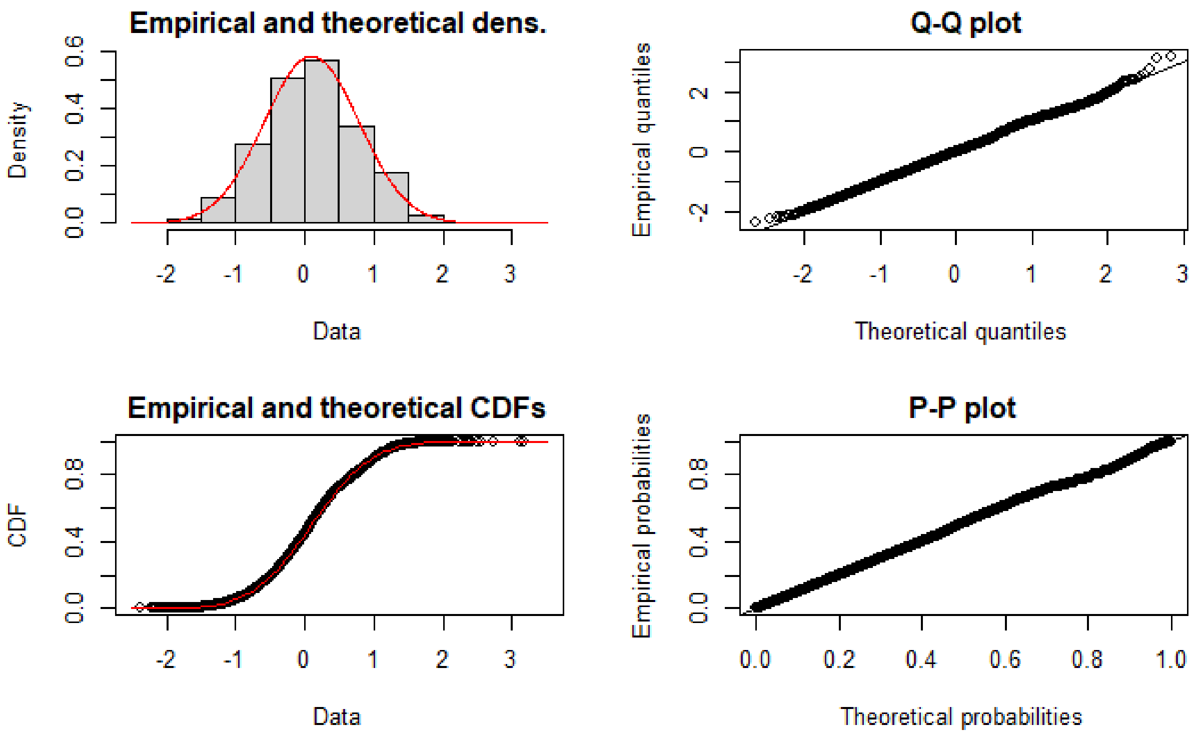

Figure S7: Goodness of fit plots for normal distribution fitting of all household lead sampling data.

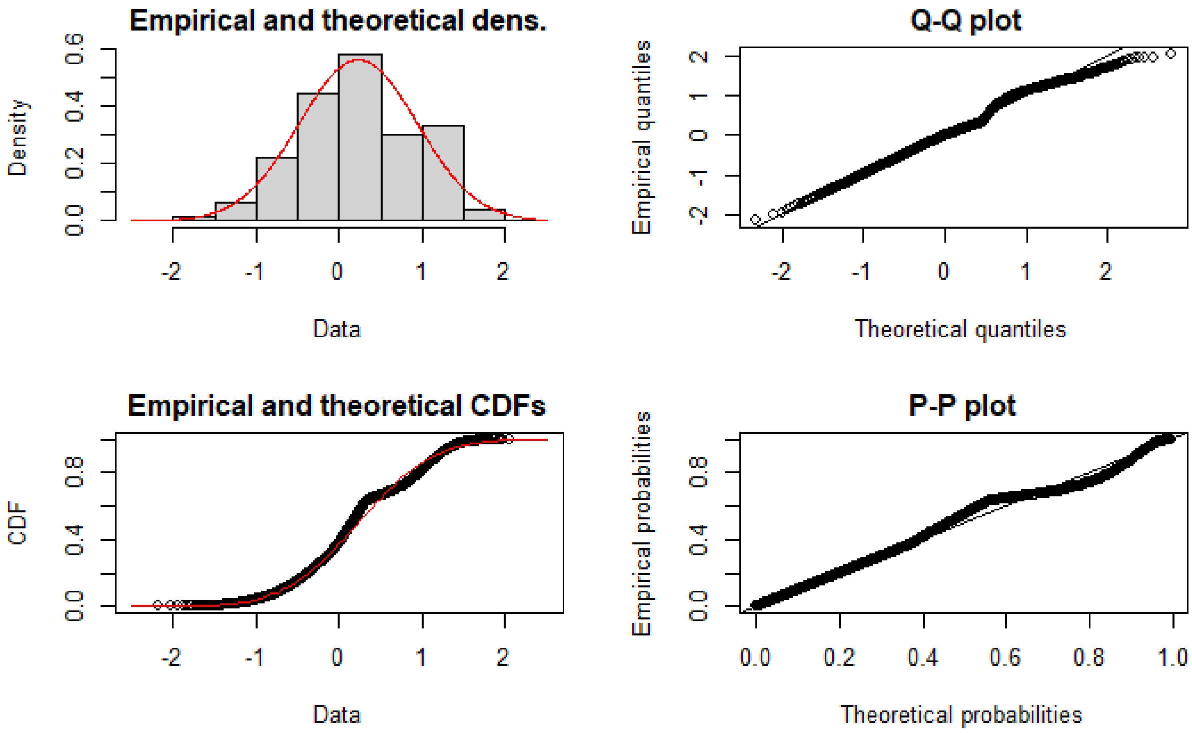

Figure S8: Goodness of fit plots for normal distribution fitting of 2016 household lead sampling data.

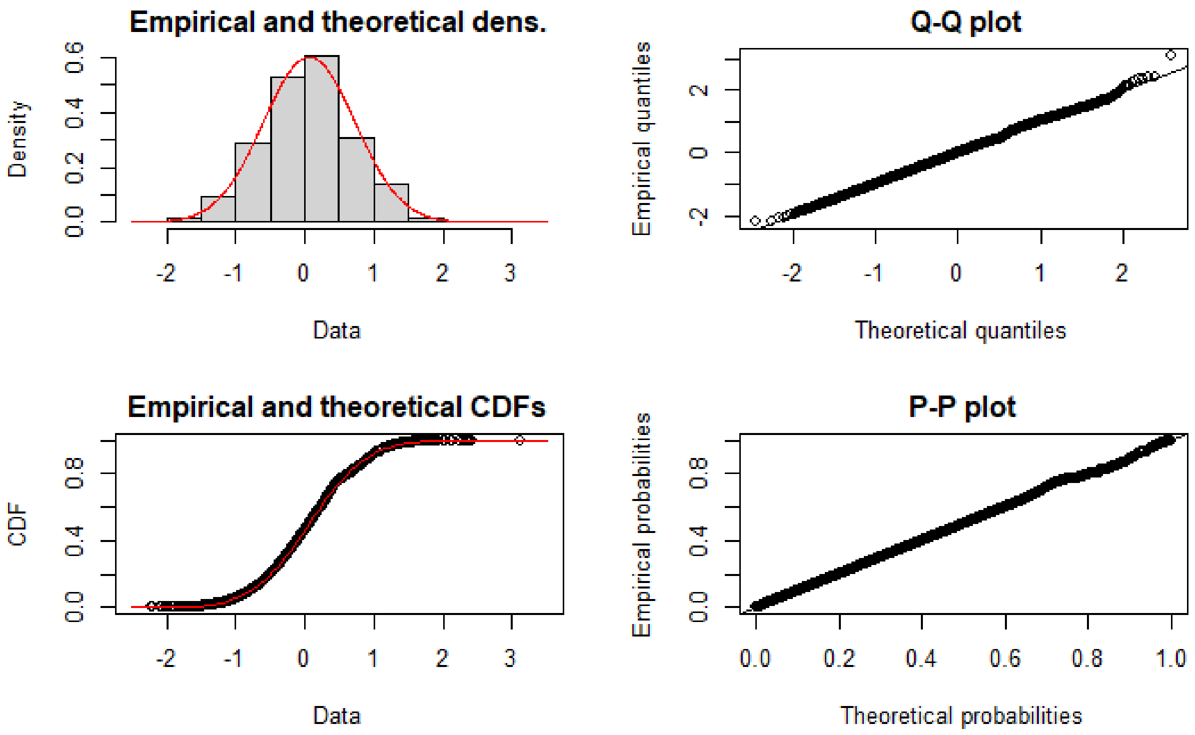

Figure S9: Goodness of fit plots for normal distribution fitting of 2017 household lead sampling data.

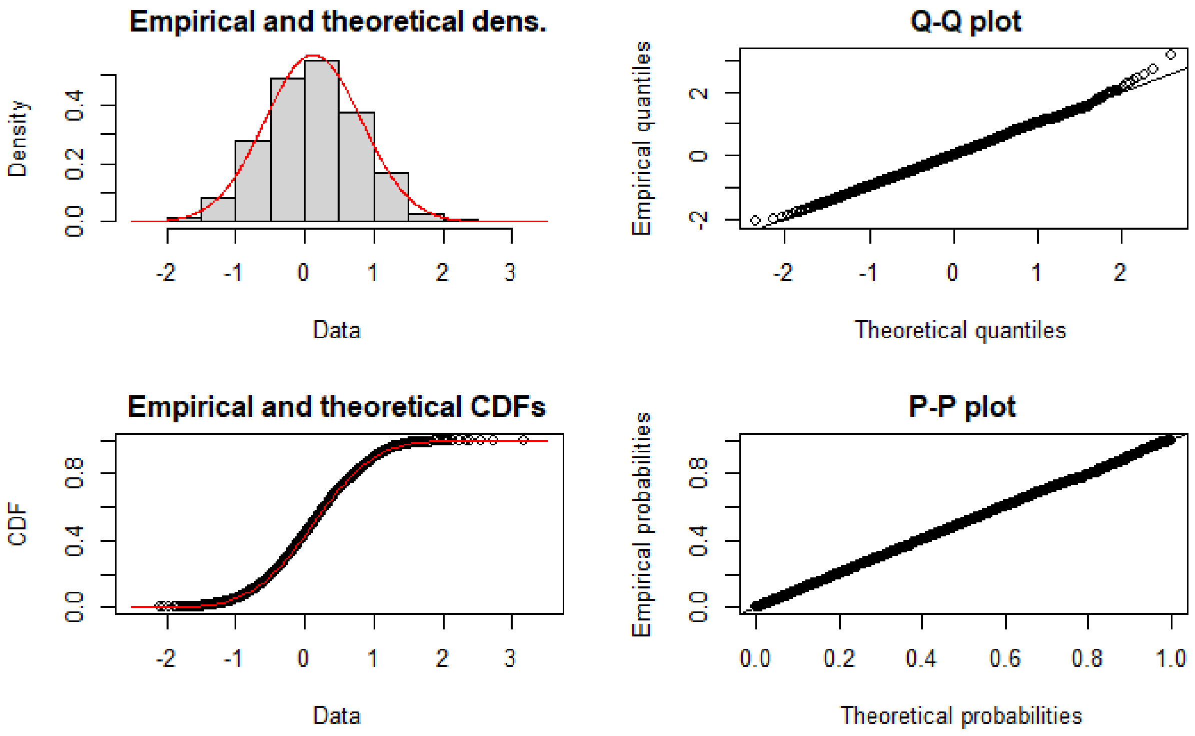

Figure S10: Goodness of fit plots for normal distribution fitting of 2018 household lead sampling data.

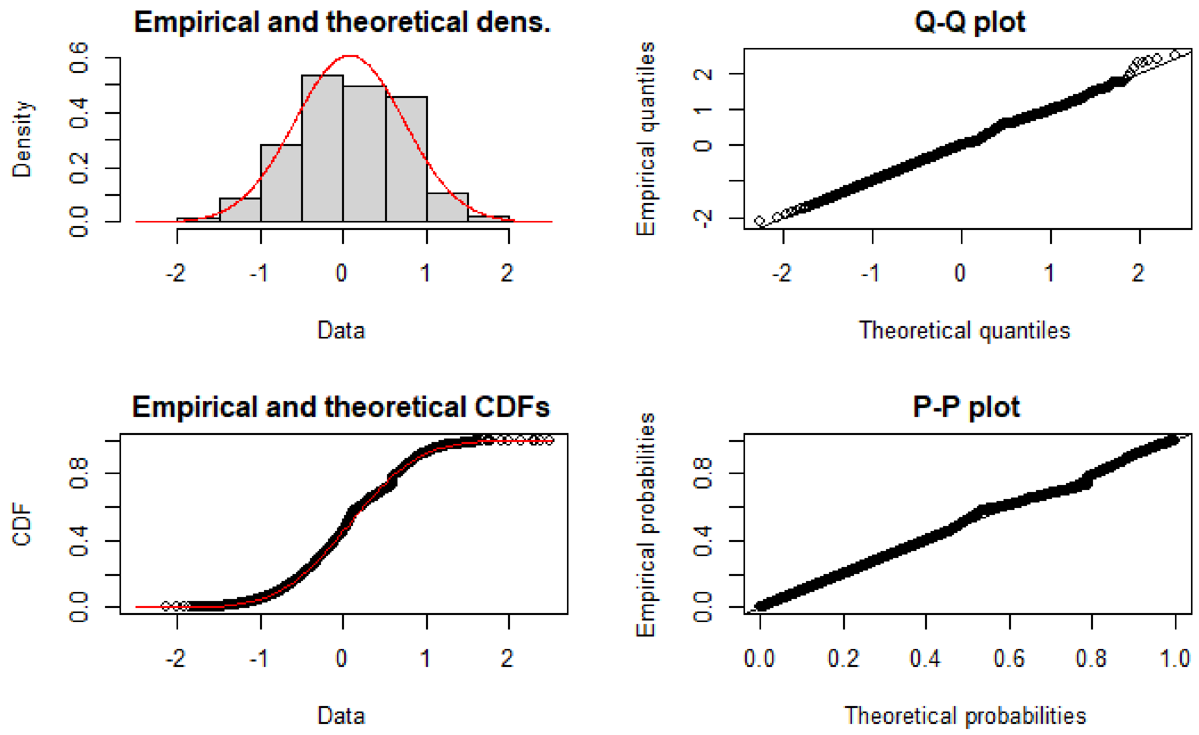

Figure S11: Goodness of fit plots for normal distribution fitting of 2019 household lead sampling data

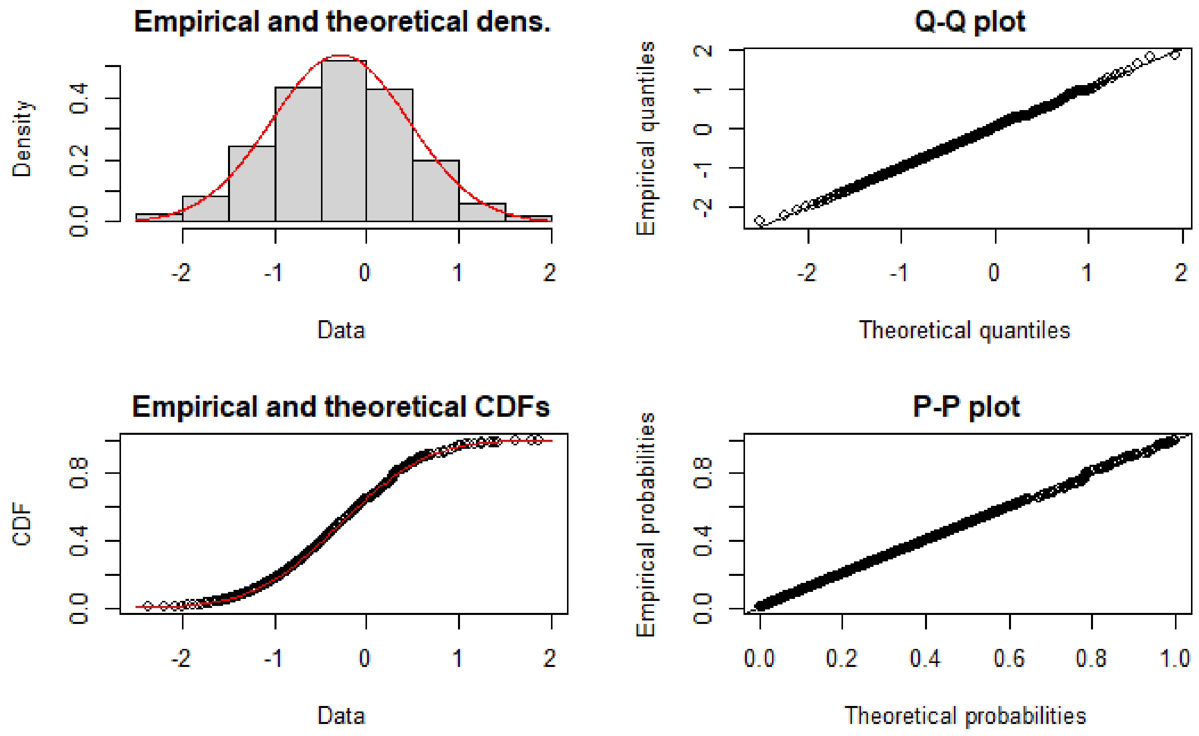

Figure S12: Goodness of fit plots for normal distribution fitting of 2020 household lead sampling data

Table S11: Parameters and goodness of fit statistics for fitted normal distribution to log-transformed household lead sampling data

Customer-requested data Grouping	Goodness of Fit Test Statistic				Normal Parameters	
	Lilliefors Test	KS Test	BIC	AIC	Mean	Standard Deviation
All Customer-requested data (2016-2020)	0.02	**rejected**	29829	29814	0.10	0.69
2016	0.07	**rejected**	6275	6263	0.23	0.71
2017	0.03	**rejected**	12788	12774	0.06	0.66
2018	0.02	not rejected	4924	4913	0.12	0.70
2019	0.05	**rejected**	4827	4839	0.07	0.66
2020	0.02	not rejected	789	781	-0.29	0.74

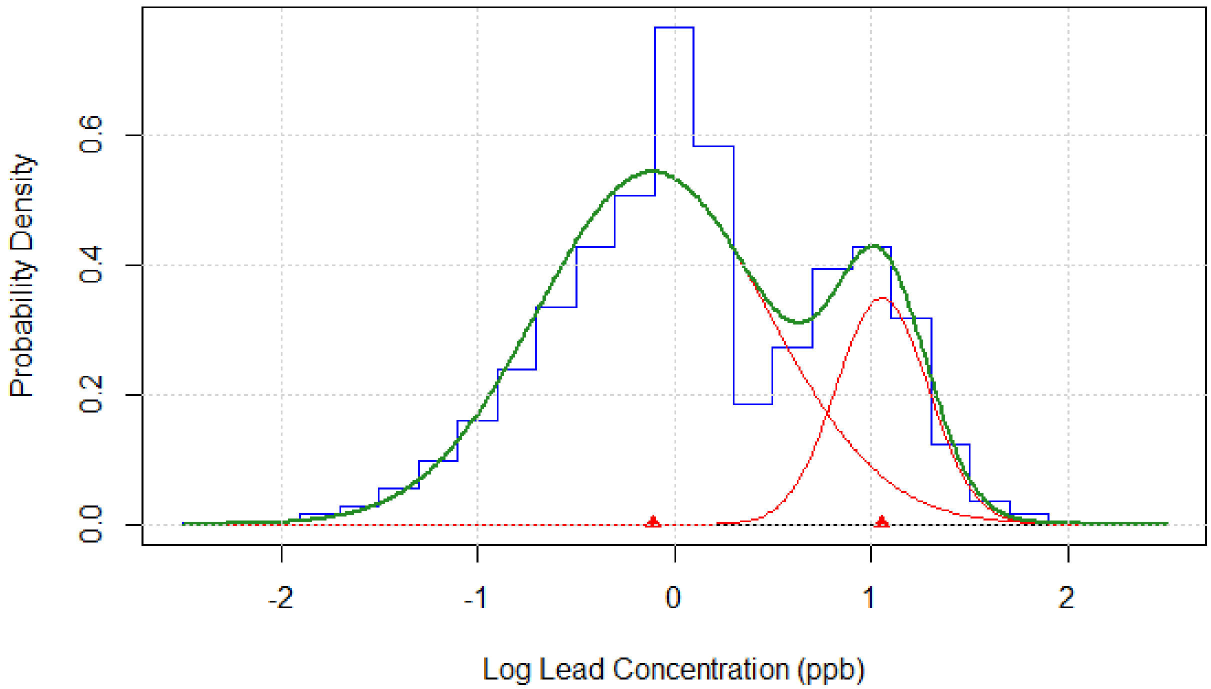

Figure S13: Probability density plot of mixture distributions fit to 2016 Customer Requested lead concentration data. The original data are shown in blue. The individual fitted distributions are shown in red, and the combined fitted distribution is shown in green.

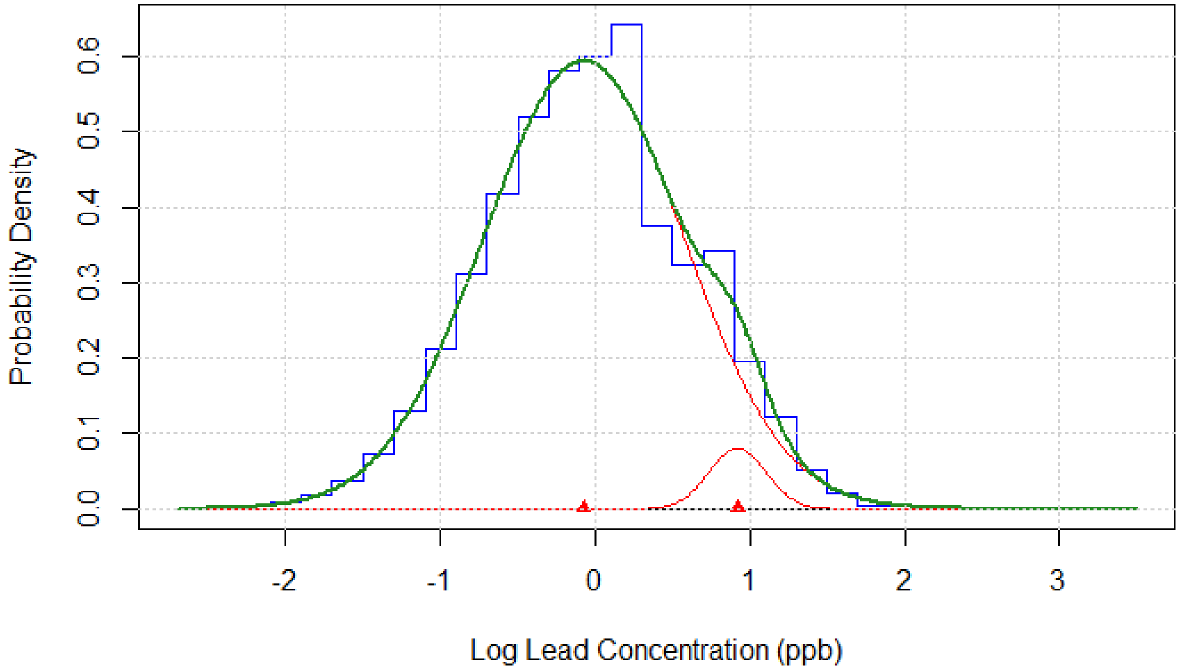

Figure S14: Probability density plot of Mixture distributions fit to 2017 Customer Requested lead concentration data. The original data are shown in blue. The individual fitted distributions are shown in red, and the combined fitted distribution is shown in green.

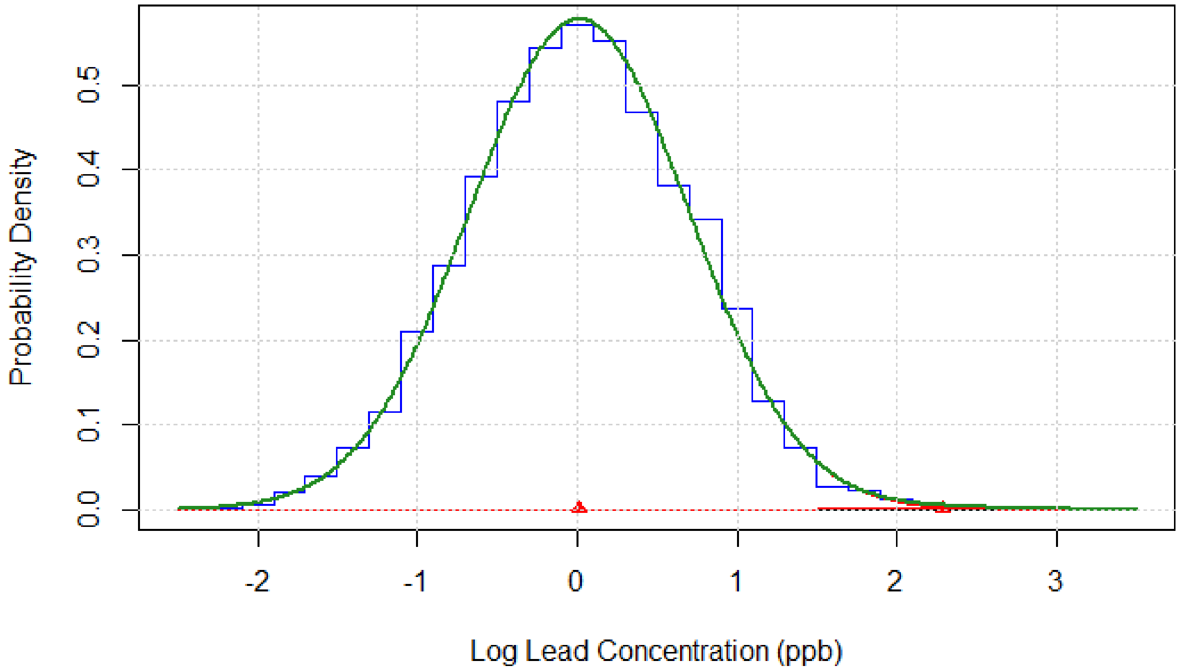

Figure S15: Probability density plot of Mixture distributions fit to 2018 Customer Requested lead concentration data. The original data are shown in blue. The individual fitted distributions are shown in red, and the combined fitted distribution is shown in green.

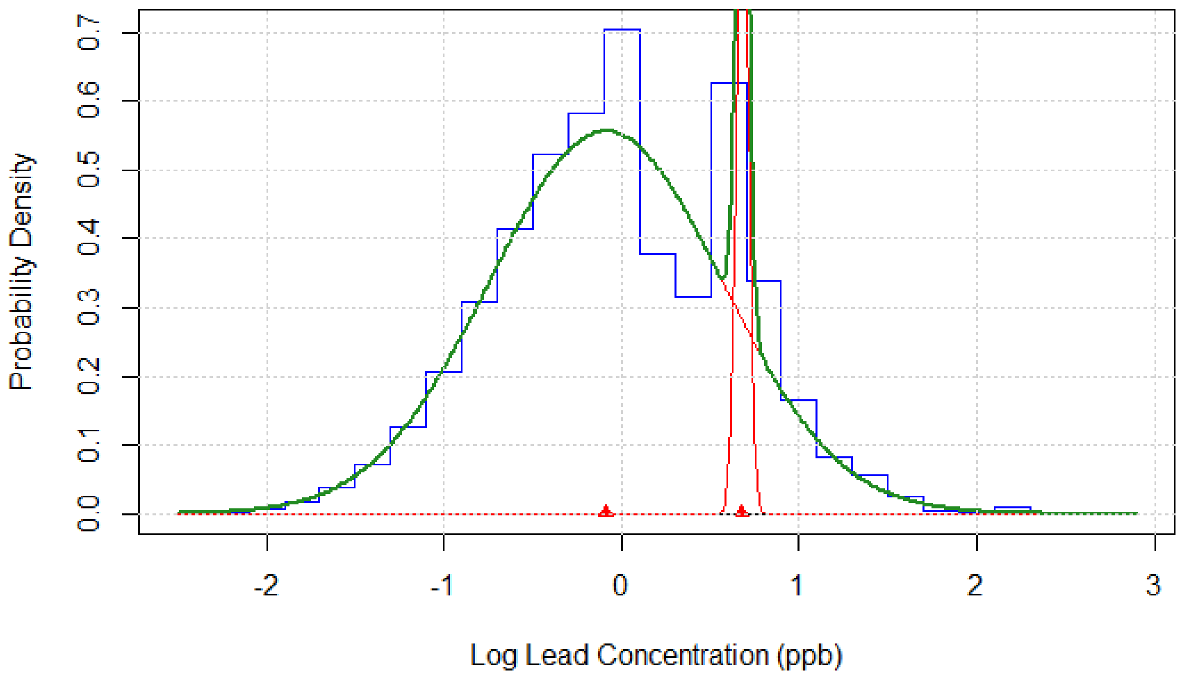

Figure S16: Probability density plot of Mixture distributions fit to 2019 Customer Requested lead concentration data. The original data are shown in blue. The individual fitted distributions are shown in red, and the combined fitted distribution is shown in green.

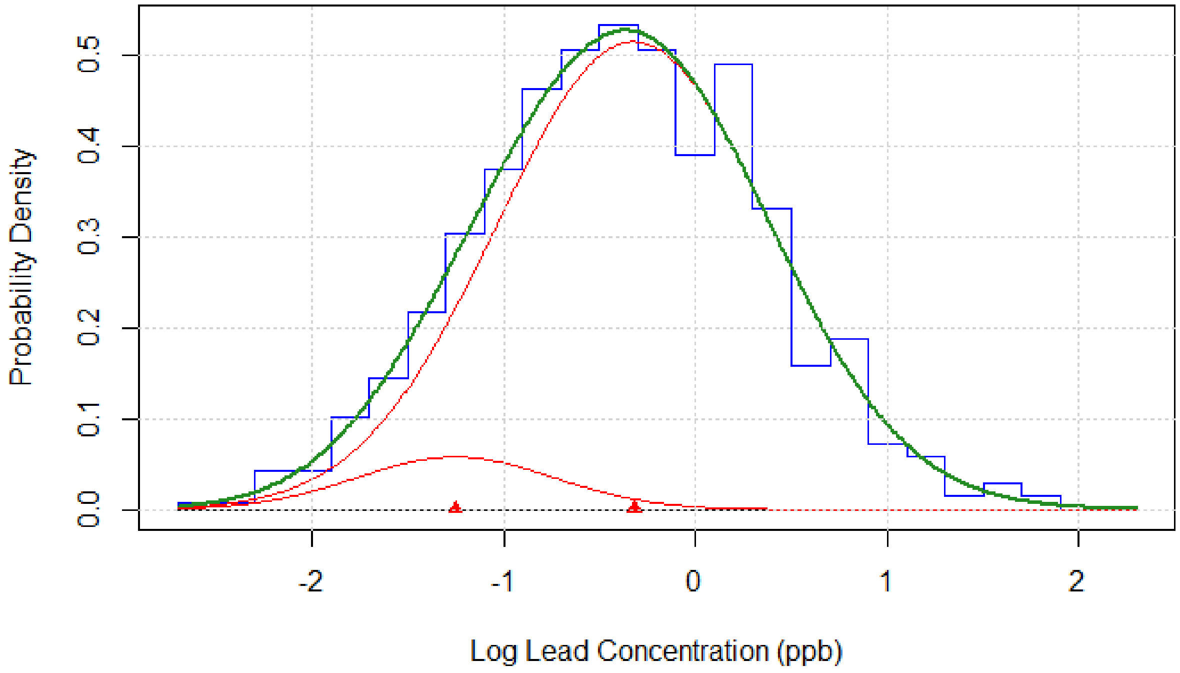

Figure S17: Probability density plot of Mixture distributions fit to 2020 Customer Requested lead concentration data. The original data are shown in blue. The individual fitted distributions are shown in red, and the combined fitted distribution is shown in green.

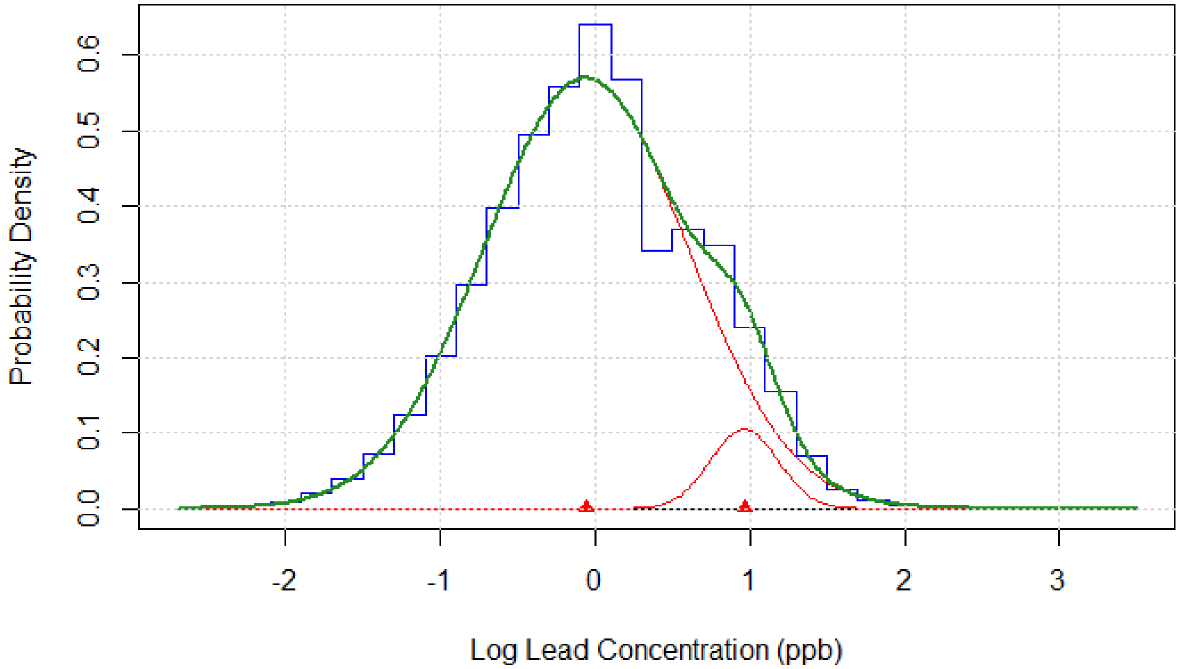

Figure S18: Probability density plot of Mixture distributions fit to all Customer Requested lead concentration data (2016-2020). The original data are shown in blue. The individual fitted distributions are shown in red, and the combined fitted distribution is shown in green.

Table S12: Parameters and goodness of fit statistics for fitted mixture distributions to log-transformed household lead sampling data.

Customer-requested data Grouping	Mixture Distribution Parameters						Goodness of Fit Test Statistic		
	Lower Dist. Mean	Lower Dist. Standard Deviation	Lower Dist. Mixture Weight	Higher Dist. Mean	Higher Dist. Standard Deviation	Higher Dist. Mixture Weight	KS	KS Test	BIC
All Customer-requested data	0.02	0.63	0.95	0.99	0.21	0.05	0.02	**rejected**	29847
2016	-0.11	0.58	0.80	1.05	0.23	0.20	0.07	not rejected	6031
2017	-0.07	0.65	0.96	0.92	0.18	0.04	0.03	not rejected	12814
2018	0.01	0.69	1.00	2.29	0.42	0.00	0.02	not rejected	4948
2019	-0.09	0.66	0.92	0.68	0.03	0.08	0.05	not rejected	4861
2020	-1.25	0.51	0.07	-0.32	0.72	0.93	0.02	not rejected	806

C. *System-Wide Lead Concentration Assessment: Customer-requested Lead Concentrations*

Comparison of customer requested samples was done by year; pairwise Kolmogorov-Smirnov test statistics are provided in **Table S13** and indicate the distribution of lead concentrations sampled at households are statistically significantly different by year. Pairwise Wilcox-Signed Rank test statistics shown **Table S*14*** in indicate that the distribution mean ranks are statistically significantly different (p <0.001) except for the comparison of 2017 to 2019 (p < 0.83).

Table S13: Results of pairwise Kolmogorov-Smirnov test for statistically significant differences in lead concentration distributions among customer-requested sampling data by year. Alpha values below 0.05, bolded below, indicate that there is enough evidence to reject the null hypothesis that the sample distributions are the same.

Year	2016	2017	2018	2019	2020
**2016**	1.00	**0.00**	**0.00**	**0.00**	**0.00**
**2017**	**0.00**	1.00	**0.00**	**0.00**	**0.00**
**2018**	**0.00**	**0.00**	1.00	**0.00**	**0.00**
**2019**	**0.00**	**0.00**	**0.00**	1.00	**0.00**
**2020**	**0.00**	**0.00**	**0.00**	**0.00**	1.00

Table S14: Pairwise Wilcoxon rank sum test statistics to test for statistically significant differences in lead concentration means for each year of household sampling. Alpha values are provided, any value below 0.05, bolded, indicates that there is enough evidence to reject the null hypothesis that the sample mean ranks are the same.

Year	2016	2017	2018	2019
**2017**	**0.00**	-	-	-
**2018**	**0.00**	**0.00**	-	-
**2019**	**0.00**	0.83	**0.01**	-
**2020**	**0.00**	**0.00**	**0.00**	**0.00**

D. *System-Wide Corrosion Control Assessment: Household and LCR Compliance Concentrations*

Kolmogorov Smirnov tests and Wilcoxon rank sum tests were conducted to compare the distribution of LCR and household lead concentration distributions by year. The test statistics, shown in **Table S15** and **Table S16**, indicate that every corresponding year of data for the LCR and customer-requested data are statistically significantly different. LCR concentration distributions are higher than those measured in the customer-requested data set.

Table S15: Pairwise Kolmogorov-Smirnov test results for the LCR and customer-requested data by year. Alpha values are provided and any value below 0.05 is bolded and indicates that there is enough evidence to reject the null hypothesis that the two datasets come from the same distribution.

Year and Data Source	2016 LCR	2017 LCR	2018 LCR	2019 LCR	2020 LCR
**2016 Customer Requested**	**0.00**	**0.00**	**0.00**	**0.00**	**0.00**
**2017 Customer Requested**	**0.00**	**0.00**	**0.00**	**0.00**	0.09
**2018 Customer Requested**	**0.00**	**0.00**	**0.00**	**0.00**	**0.02**
**2019 Customer Requested**	**0.00**	**0.00**	**0.00**	**0.00**	**0.01**
**2020 Customer Requested**	**0.00**	**0.00**	**0.00**	**0.00**	**0.00**

Table S16: Pairwise Wilcoxon rank sum test results for the LCR and customer-requested data by year. Alpha values are provided and any value below 0.05 is bolded and indicates that there is enough evidence to reject the null hypothesis that the two datasets come from the same distribution.

Year and Data Source	2016 LCR	2017 LCR	2018 LCR	2019 LCR	2020 LCR
**2016 Customer Requested**	**0.00**	**0.00**	**0.00**	**0.00**	**0.04**
**2017 Customer Requested**	**0.00**	**0.00**	**0.00**	**0.00**	0.49
**2018 Customer Requested**	**0.00**	**0.00**	**0.00**	**0.00**	0.70
**2019 Customer Requested**	**0.00**	**0.00**	**0.00**	**0.00**	0.55
**2020 Customer Requested**	**0.00**	**0.00**	**0.00**	**0.00**	**0.00**

E. *Pre- and Post-Orthophosphate Data*

An analysis of changes to the observed lead distributions in the two datasets was conducted after the introduction of orthophosphate into the drinking water system. The data were separated into pre-orthophosphate data and post-orthophosphate data for each of the data sources. LCR compliance data reported in June 2019 was not included in this analysis since the samples collected within this reporting period could include both samples from before and after orthophosphate was introduced. Household samples considered pre-orthophosphate includes data with sample analysis dated from 2016 to March 2019 and post includes data from June 2019 through June 2020.

Figures S19 to S22 show the single normal distribution fit to the log transformed LCR compliance and household data from before and after the introduction of orthophosphate, and **Table S17** provides goodness of fit statistics and the normal parameters. The qualitative assessment of the figures and goodness of fit statistics indicate that LCR pre- and post-orthophosphate data fit a single distribution well. Two component mixture models were assessed for the pre- and post-orthophosphate household lead concentration data, as shown in **Figure S23 and Figure S24**. **Table S18** provides goodness of fit statistic and relevant model parameters. Qualitative and quantitative assessment indicates that the pre-orthophosphate data have a slight improvement in fit with the mixture model. The post-orthophosphate data exhibit a considerably better fit with the mixture model.

Pairwise Kolmogorov-Smirnov tests were performed to determine if statistically significant differences in the fitted distributions of the two datasets (LCR and household) existed pre- and post-orthophosphate. Results of the pairwise tests are included as **Table S19** and **Table S20** for pre- and post-orthophosphate data, respectively. The tests indicate that mixture

distribution components from the customer requested data and the single distribution of the LCR compliance data are all statistically significantly different from each other before and after the introduction of orthophosphate. Pairwise Wilcoxon rank sum tests indicate that all mean ranks are also statistically significantly different before and after orthophosphate ($p < 0.001$).

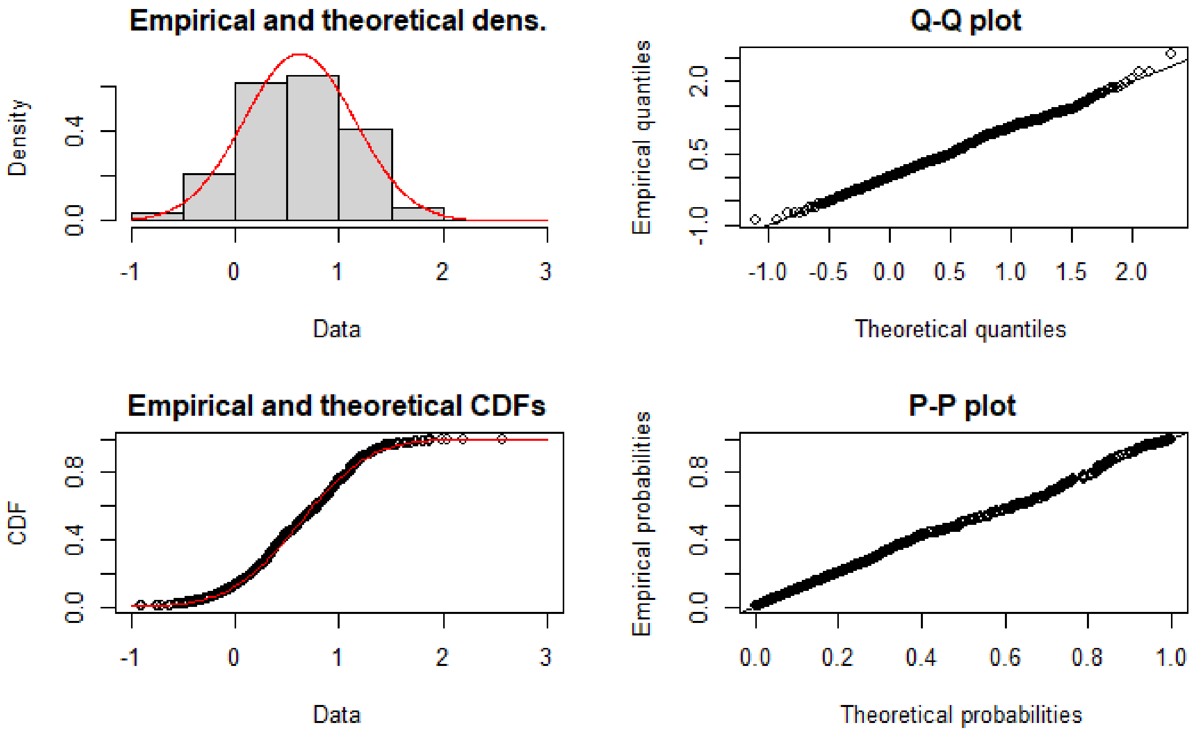

Figure S19: Goodness of fit plots for normal distribution fitting of pre-orthophosphate LCR Compliance sampling data.

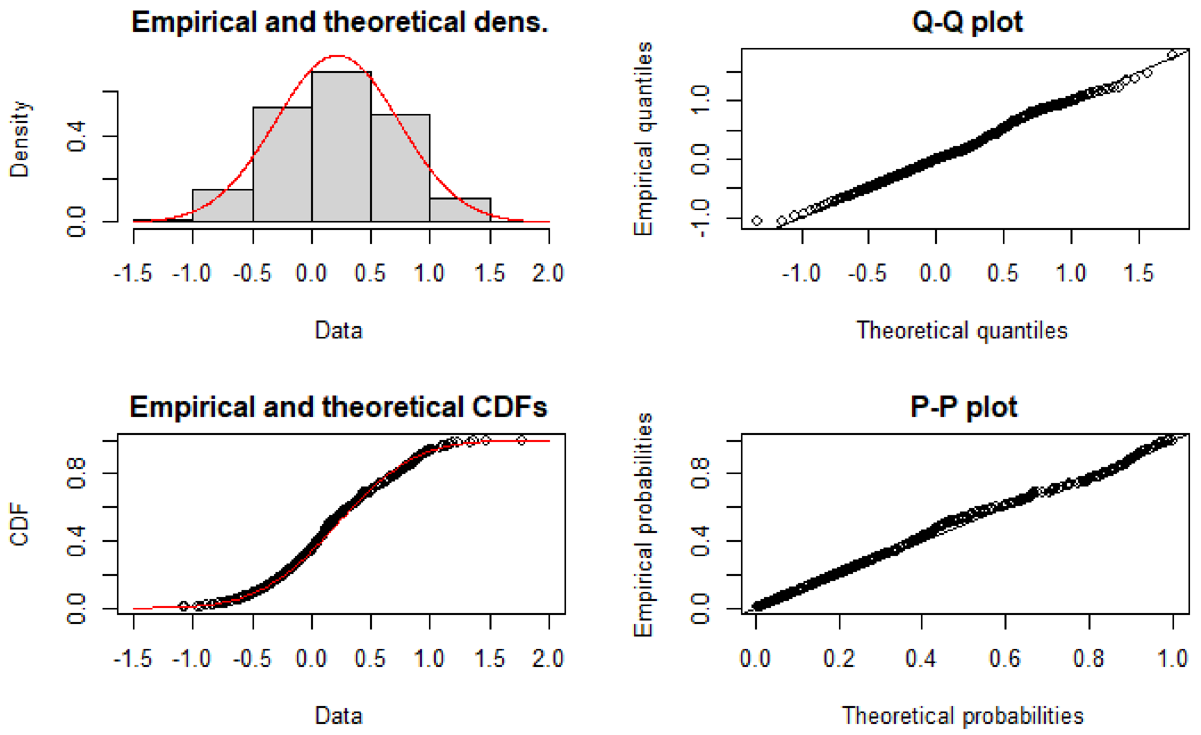

Figure S20: Goodness of fit plots for normal distribution fitting of post-orthophosphate LCR Compliance sampling data.

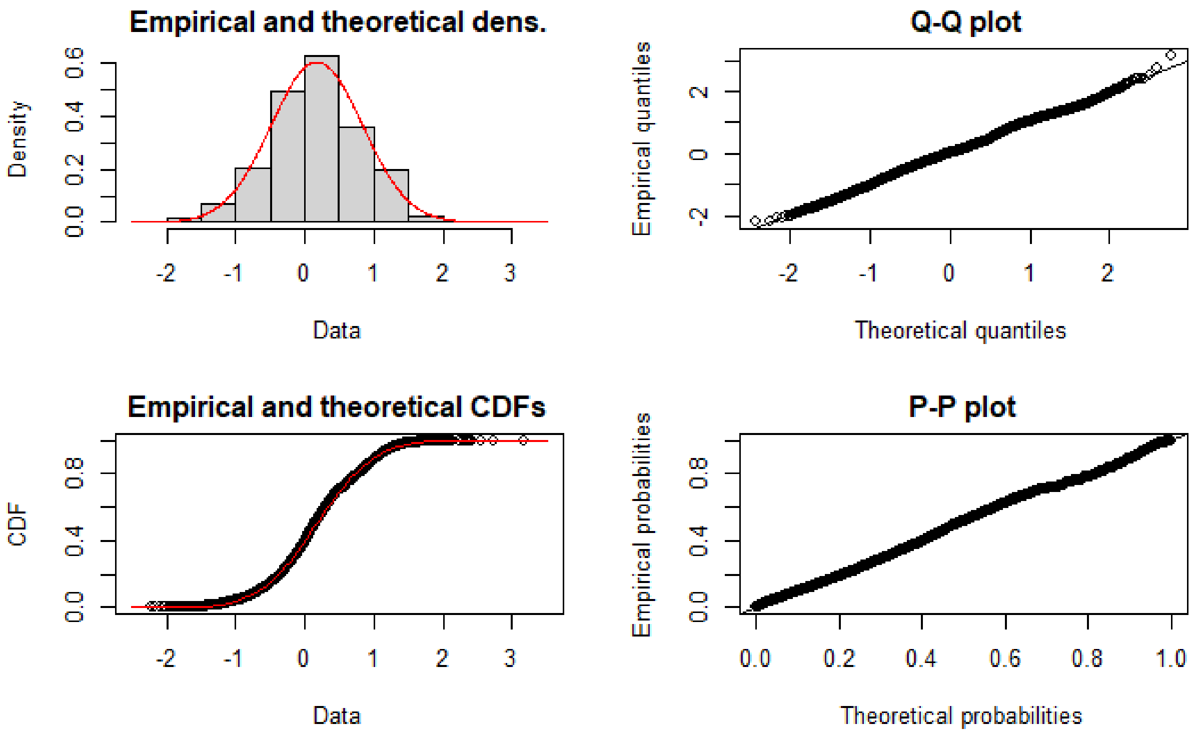

Figure S21: Goodness of fit plots for normal distribution fitting of pre-orthophosphate household lead sampling data.

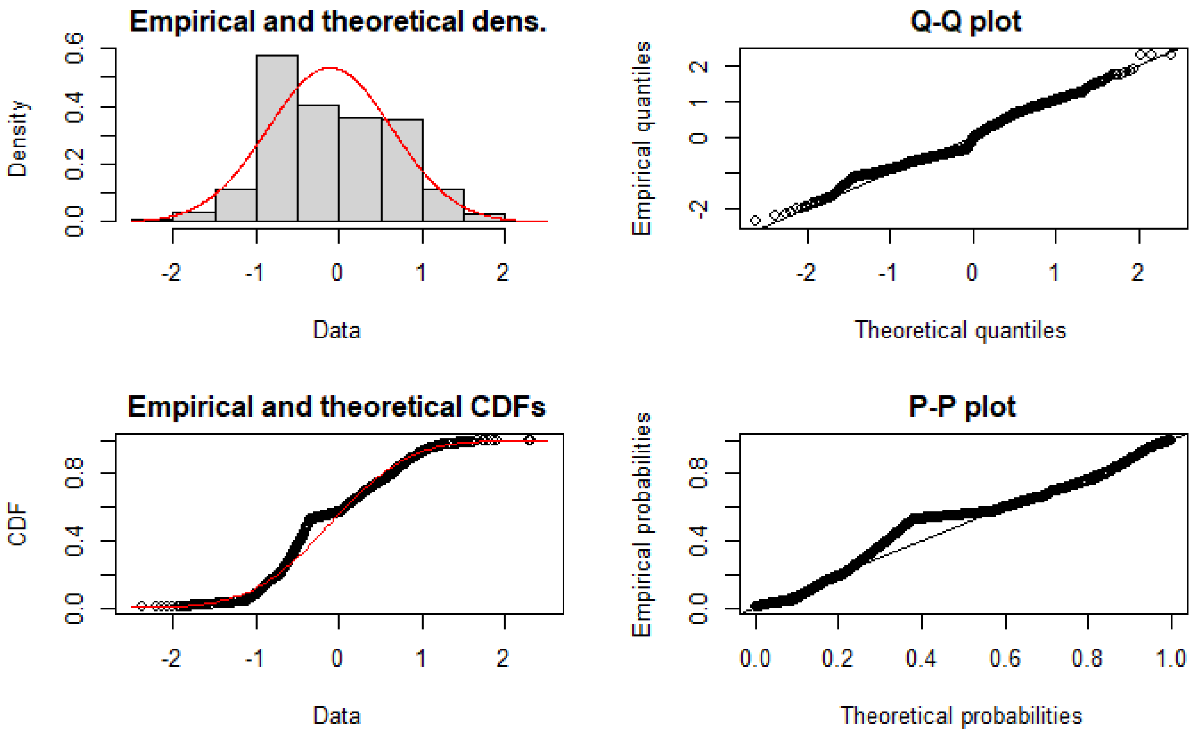

Figure S22: Goodness of fit plots for normal distribution fitting of post-orthophosphate household lead sampling data.

Table S17: Parameters and goodness of fit statistics for fitted normal distribution to log-transformed lead sampling data grouped by dates that correspond to before and after the introduction of orthophosphate for corrosion control.

Remediation Stage	Data Source	Goodness of Fit Test Statistic				Normal Parameters	
		Lilliefors Test	KS Test	BIC	AIC	Mean	Standard Deviation
Pre-Orthophosphate	LCR Compliance (every six-months) (June 2016 – Dec 2018	0.05	not rejected	1231	1222	0.61	0.53
	Household (2016 – March 2019)	0.03	**rejected**	22501	22486	0.17	0.66
Post-Orthophosphate	LCR Compliance (every six-months) (Dec 2019 – June 2020)	0.05	not rejected	510	502	0.21	0.52
	Household (June 2019- June 2020)	0.14	**rejected**	2767	2756	-0.11	0.75

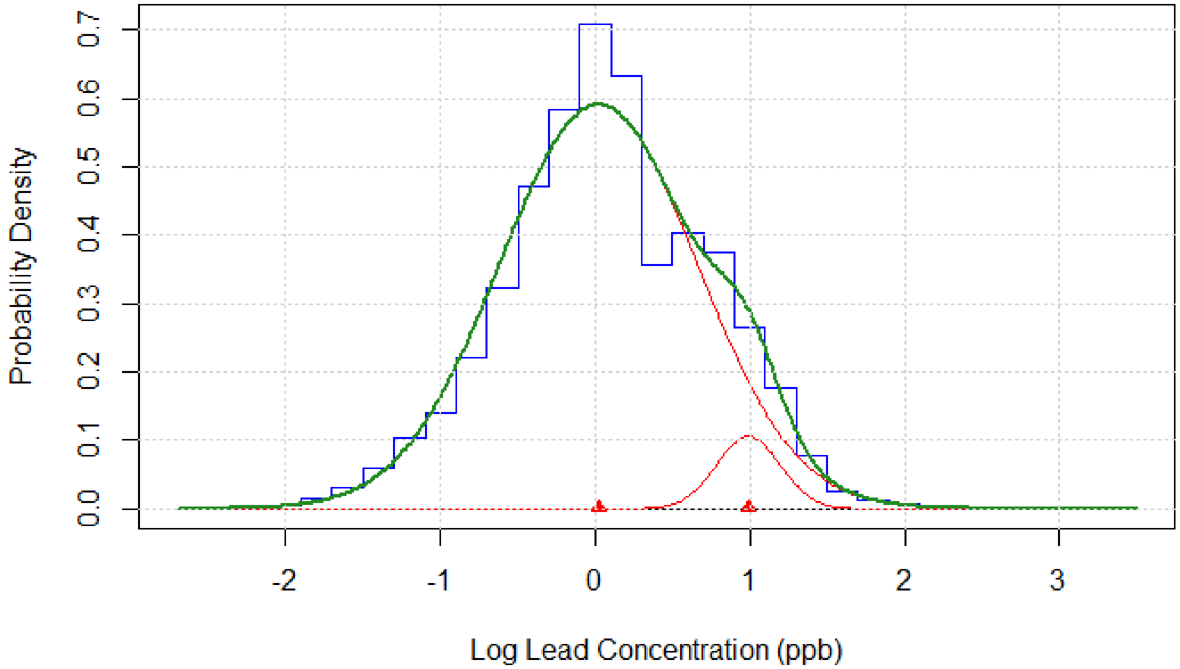

Figure S23: Probability density plot of Mixture distributions fit to pre-orthophosphate introduction (2016-2018) Customer Requested lead concentration data. The original data are shown in blue. The individual fitted distributions are shown in red, and the combined fitted distribution is shown in green.

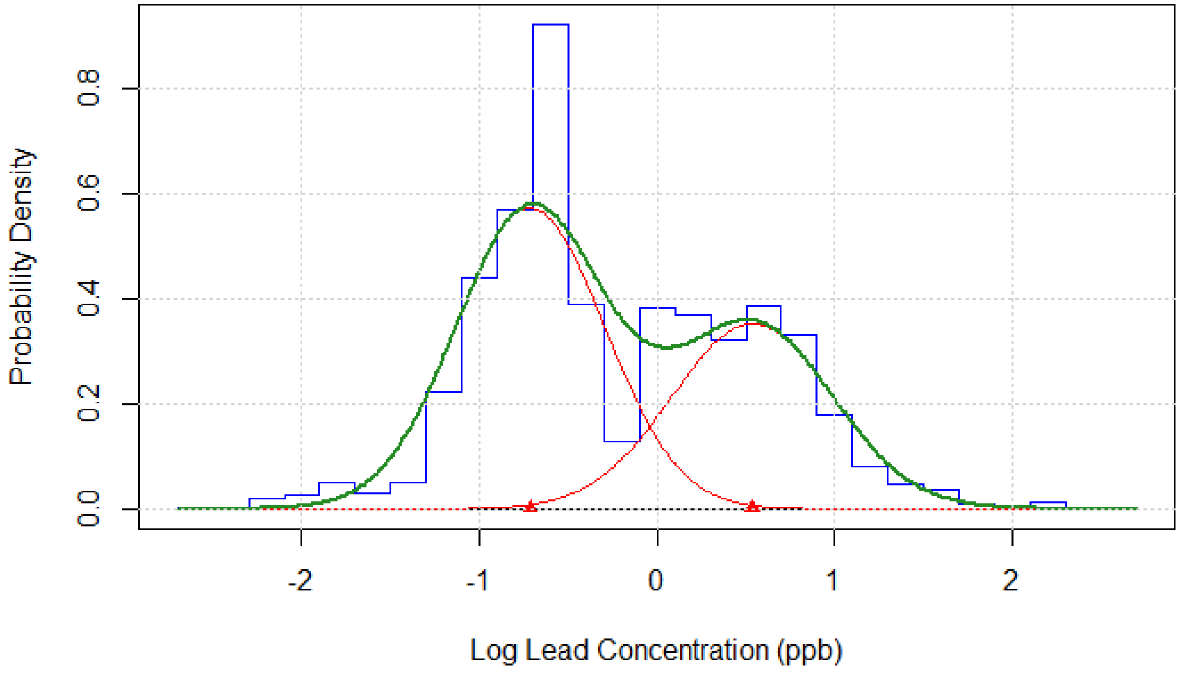

Figure S24: Probability density plot of Mixture distributions fit to post-orthophosphate introduction (2019-2020) Customer Requested lead concentration data. The original data are shown in blue. The individual fitted distributions are shown in red, and the combined fitted distribution is shown in green.

Table S18: Parameters and goodness of fit statistics for fitted mixture distributions to log-transformed pre- and post-orthophosphate household lead sampling data.

Customer-requested data Grouping	Mixture Distribution Parameters						Goodness of Fit Test Statistic		
	Lower Dist. Mean	Lower Dist. Standard Deviation	Lower Dist. Mixture Weight	Higher Dist. Mean	Higher Dist. Standard Deviation	Higher Dist. Mixture Weight	KS	KS Test	BIC
Pre-Orthophosphate	0.02	0.64	0.95	0.99	0.21	0.05	0.03	not rejected	22531
Post-Orthophosphate	-0.88	0.40	0.60	0.44	0.46	0.40	0.14	not rejected	2661

Table S19: Results of pairwise Kolmogorov-Smirnov test for statistically significant differences in lead concentration distributions for the fitted distributions for all pre-orthophosphate data (2016- March 2019). Alpha values below 0.05, bolded below, indicate that there is enough evidence to reject the null hypothesis that the sample distributions are the same.

**Fitted Distribution**	**Component 1**	**Component 2**	**LCR Compliance Data**
**Component 1**	1.00	**0.00**	**0.00**
**Component 2**	**0.00**	1.00	**0.00**
**LCR Compliance Data**	**0.00**	**0.00**	1.00

Table S20: Results of pairwise Kolmogorov-Smirnov test for statistically significant differences in lead concentration distributions for the fitted distributions for all post-orthophosphate data (2019-2020). Alpha values below 0.05, bolded, indicate that there is enough evidence to reject the null hypothesis that the sample distributions are the same.

**Fitted Distribution**	**Component 1**	**Component 2**	**LCR Compliance Data**
**Component 1**	1.00	**0.00**	**0.00**
**Component 2**	**0.00**	1.00	**0.00**
**LCR Compliance Data**	**0.00**	**0.00**	1.00

F. *Monte Carlo Simulations*

Monte Carlo sampling of the fitted customer requested distributions pre- and post-orthophosphate and by year was performed to compare the resulting 90th percentile concentration from the customer-requested households to the reported 90th percentile concentration from the LCR compliance data. The number of samples per iteration was selected as 150 to represent the number of samples typically reported by the utility per compliance sampling period. The number of iterations was determined to be 10,000 based on convergence of the mean for the sampled iterations. Convergence is considered the number of iterations at which the cumulative mean no longer varies as additional sampling iterations are added. **Figure S25** and **Figure S26** shows convergence of the simulation as the number of iterations increases. For pre- and post-orthophosphate and each year, the cumulative mean of the sampled iterations is calculated as the number of iterations increases. The mean stabilizes around 2000 iterations, after which little variation is observed. Thus, 10,000 iterations are considered sufficient based on this analysis.

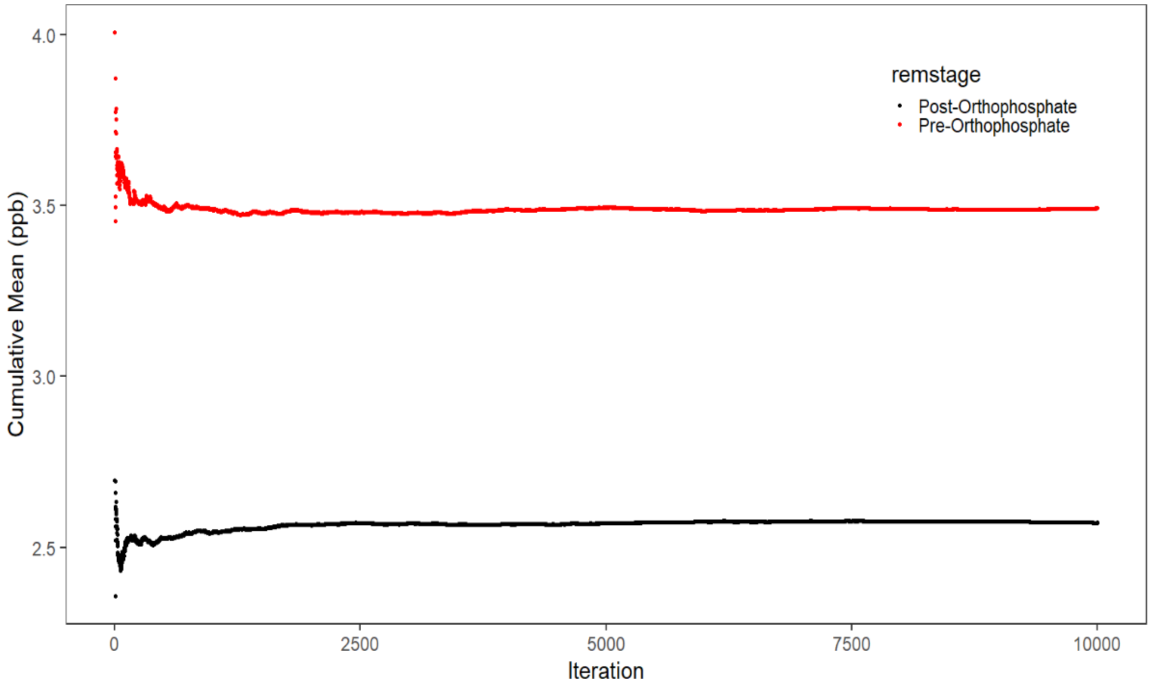

Figure S25: Variation of the mean of the sampled lead concentrations for each year of Customer-requested lead sampling data.

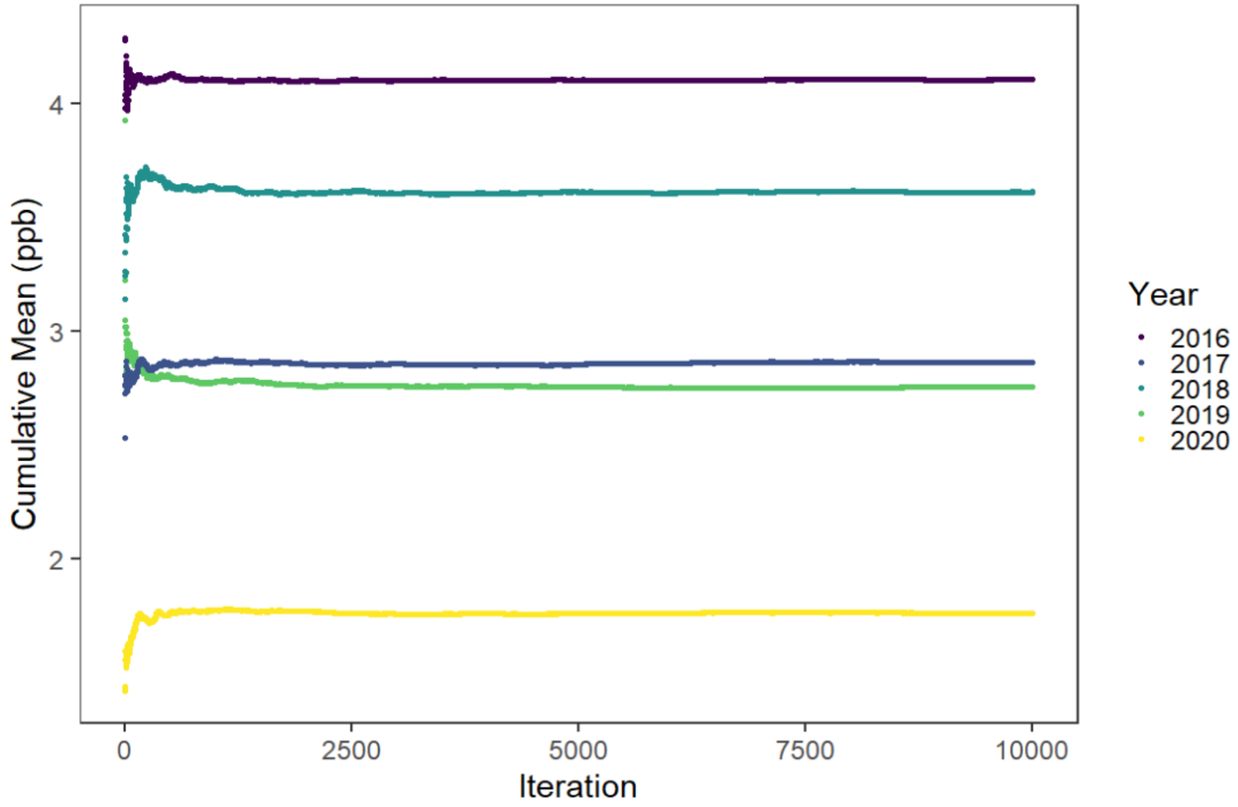

Figure S26: Variation of the mean of the sampled lead concentrations for each year of Customer-requested lead sampling data.

G. *Spatial customer-requested bias assessment*

The publicly available customer-requested data include masked geographic information including the block number and street name for each data point collected. ZIP code boundary information is made publicly available on the Allegheny County Open GIS data website.[12,13] ArcGIS's geocoding tools were used to geolocate each of the masked customer-requested data points.

The customer-requested data points were binned to their respective ZIP codes and an analysis of the fraction of data points above the reporting limit was conducted. Information regarding the number of homes in each ZIP code was obtained from the US Census website.[14] This information was then used to determine the approximate fraction of homes sampled in each ZIP code. Based on the anonymization of the data, it is not possible to determine if the same home was sampled multiple times since location information from the customer-requested data was masked. This analysis assumes that samples are independent. It is important to note that if a home tested above the 15-ppb action limit, PWSA would reach out to the customer directly to determine the source of lead and provide guidance on reducing lead concentrations, so subsequent sampling, if necessary, would be performed by the utility.[15]

IV. Results and Discussion

A. *System Wide Lead Concentrations: Customer Requested Concentration Data*

i. Customer-Requested lead concentration data from 2016 to 2020

The customer-requested (household) lead testing program was begun in February 2016, shortly before the exceedance of the Action Limit was reported. PWSA provided concerned consumers with a one-liter bottle to self-collect a first-draw sample after a 6-hour stagnation period. Samples were mailed to one of three partnered laboratories for testing. If tested lead concentrations were above 10 ppb, the utility would provide follow-up information, and customers were offered a drinking water filter.

Figure S27 shows a temporal histogram of the customer requested sampling frequency observed from 2016 to 2020. Following the report of elevated lead in compliance data in June 2016, customer requested sampling significantly increased with 2,274 samples submitted for testing from July through December 2016 and 5,272 samples from Jan through June 2017. Requests continued at a slower pace in 2018-2020.

Figure S28 is a scatter plot of the raw lead concentrations reported from the customer requested samples on a logarithmic scale. The clustering of data highlights the effect of the reporting limits described in **Table S2**. The scatterplot shows a decreasing trend in the lead concentrations after the introduction of orthophosphate in April 2019, with fewer concentrations reported above 10 ppb. Most samples shown on the scatterplot are below the EPA AL, indicating most households did not have elevated levels of lead in their drinking water, even during the non-compliance period when corrosion control was not optimized.

Table S21 provides summary statistics for the household lead concentration data. Only in 2016 was the 90th percentile lead concentration above the EPA AL (value of 15.8 ppb).

Following the introduction of orthophosphate, the 90th percentile concentration for customer-requested samples in 2020 was 4.16 ppb. The median lead concentrations calculated for each year were below the reporting limit, indicating that more than half of all homes tested each year had lead concentrations below the laboratory reporting limit. Median values included in **Table S21** were calculated following imputation of the below detection limit samples (as described in **Section II**)

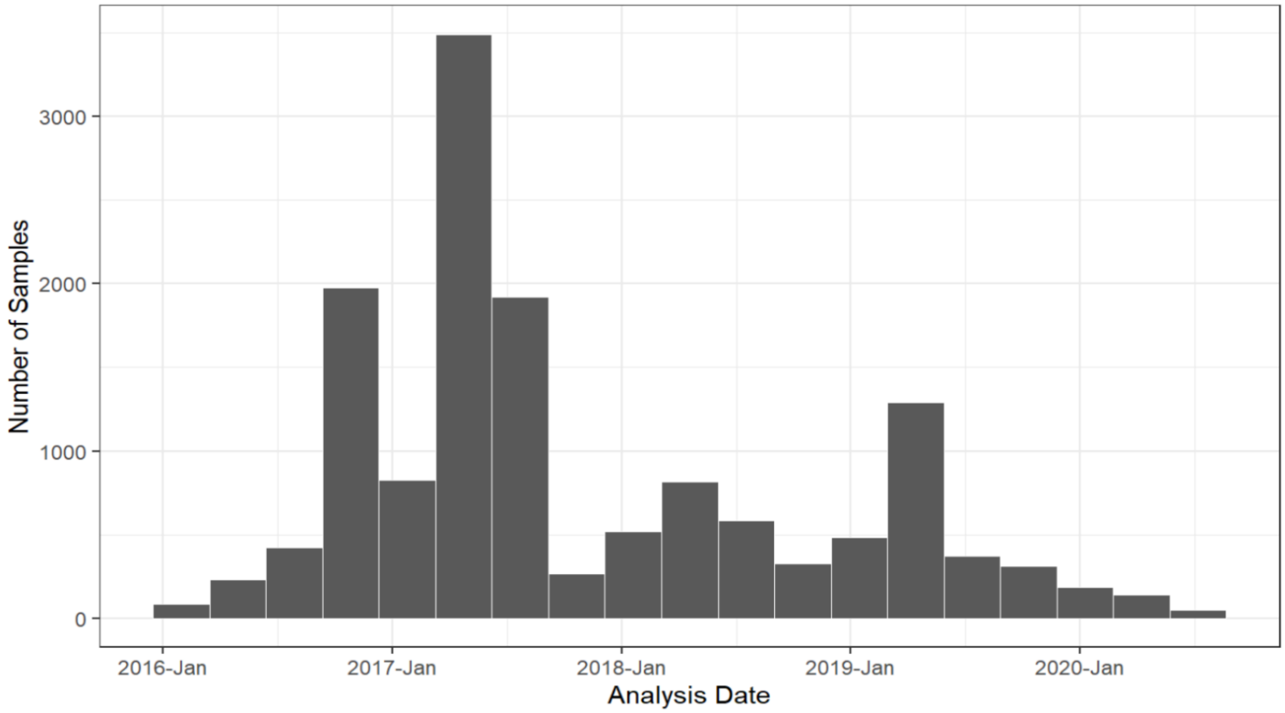

Figure S27: Temporal histogram of customer-requested lead sampling data collected from 2016 to 2020. A total of 14,293 samples were analyzed between January 2016 and June 2020.

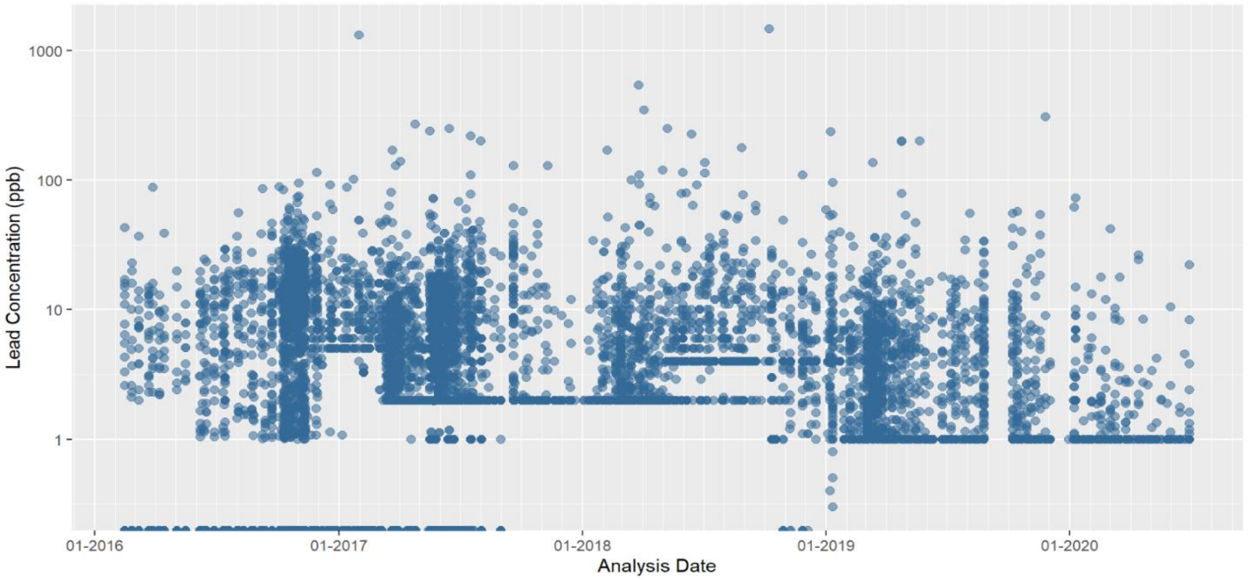

Figure S28: Scatterplot of household lead sampling results from 2016 to 2020 on a logarithmic concentration scale.

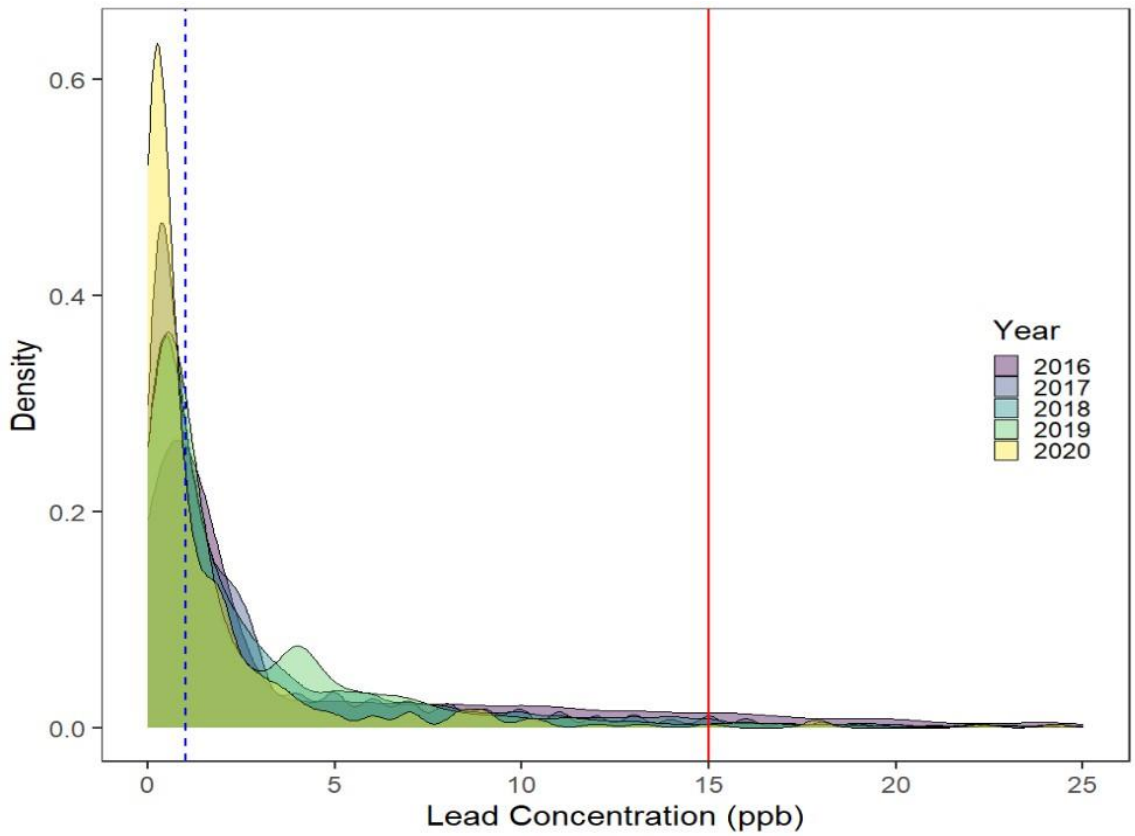

Figure S29: Density plot of household lead sampling data by year on an arithmetic scale. The solid vertical red line represents the EPA AL of 15 ppb. The blue dashed vertical line indicates the reporting limit of 1ppb. **SI Section II** provides additional information on reporting limits used in the customer-requested data.

Table S21: Summary statistics for customer-requested data by year after using ROS methods to account for samples below reporting limit.

Year	Sample size	Number of samples below reporting limit (% below RL)	Number of samples above AL	90th Percentile (ppb) following LCR method	Arithmetic mean (ppb)	Median (ppb)	Standard deviation
**All**	14293	9512 (67)	813	10.7	4.39	1.21	20.5
**2016**	2901	1623 (56)	317	15.8	5.41	1.44	9.34
**2017**	6327	4617 (73)	267	9.00	3.83	1.15	19.3
**2018**	2309	1753 (76)	128	11.0	5.64	1.31	35.9
**2019**	2410	1294 (54)	93	7.60	3.77	1.11	12.5
**2020**	347	225 (65)	8	4.16	2.17	0.51	6.40

B. *System Wide Corrosion Control Assessment: Customer Requested and LCR Compliance Data*

ii. LCR Compliance sampling lead concentrations from 2016 to 2020

Following the exceedance of the EPA Action Limit reported in June 2016, LCR compliance sampling was conducted every six months at Tier 1 sites until June 2020, when the utility came back into compliance. Tier 1 households are targeted with the purpose of determining if corrosion control is working adequately. Due to the size of the distribution network (> 100,000 connections), a minimum of 100 samples is required to determine corrosion control efficacy. The utility sends out over 100 sample requests to residents known to be living in Tier 1 households. Residents are instructed to collect the sample as a first draw one-liter sample after a six-hour stagnation period and mail the sample back to the utility for analysis. All samples returned through the LCR compliance testing program must be reported, as such, eight of the compliance periods reported during the study period had sample sizes greater than the minimum 100 samples.

Based on visual assessment of the fitted distributions and the results of the Kolmogorov-Smirnov tests (**Table S3, Figures S4 to S12**) the fitted log-normal distributions were acceptable for all years. **Figure S30** presents a notched boxplot of compliance data by year. The customer-requested data by year (blue outline) and the LCR data by year (grey outline) are shown in **Figure S31**. **Table S22** provides summary statistics for the LCR compliance sampling data by year. After the introduction of orthophosphate, 90th percentile concentrations reported in December 2019 and June 2020 are below the EPA action limit. **Figure S32** provides the probability density functions and cumulative distribution functions for all years. Both plots

indicate that following the introduction of orthophosphate the LCR compliance data distributions were mostly below the EPA AL and almost half of the samples are below the reporting limit.

The compliance data indicate that the reported 90th percentile statistic of LCR data from June 2016 to June 2019 exceeds the federal AL (15ppb) (red dot on or above red line in **Figure S30 and Figure S31**), with the exception of compliance data in June 2018 (90th percentile was 10 ppb). Lead concentration measured in December 2019 and June 2020 are significantly lower than most prior data, with 90th percentile values of 10 ppb and 5.1 ppb, indicating a return to compliance with the LCR. This was expected since a change to orthophosphate for corrosion control was initiated in April 2019. Also as expected, pairwise Wilcoxon rank sum tests and Kolmogorov-Smirnov tests indicate that samples collected in 2020 differ significantly from all the other sampling periods; previous periods were statistically significantly different from some but not all other sampling periods (see **Table S9 and Table S10**).

Despite the LCR non-compliance from 2016 through mid-2019, most sample values were below 15ppb throughout the time period. The percentile representing 15ppb in each of the years was 84 in 2016, 89 in 2017, 90 in 2018, 92 in 2019 and 99 in 2020. Also, even within compliance data, which are selected to represent locations expected to be elevated when corrosion control is ineffective due to the expected presence of lead service lines or indoor lead plumbing, 22 to 40 percent of the samples taken between June 2016 and June 2019 were below the reporting limit for lead (below 1 ppb from 2016 to 2020 and below 4 ppb for select samples in 2018). After the introduction of orthophosphate for corrosion control, approximately 40 percent of the samples were below the reporting limit of 1ppb. Outliers in **Figure S30** include values of 380 and 110 ppb in 2017; 96 ppb in 2018; and 195 ppb in 2019. In 2020, after the introduction of orthophosphate resulted in much better corrosion control, the only outlier value

was 29.7 ppb, and only 2 values reported above 15ppb. For each sampling period, all sites with sample values above 10 ppb were contacted by the utility and were offered drinking water filters.

Figure S33 provides a cumulative distribution plot of the LCR and household data by year. The LCR data are represented by the solid lines and the household data by the dashed lines. It is apparent from the figure that the customer requested data is always to the left of the LCR compliance data, indicating that the distribution of lead concentrations from the customer requested data is lower than the distribution of lead concentrations measured for the LCR compliance data for any given year.

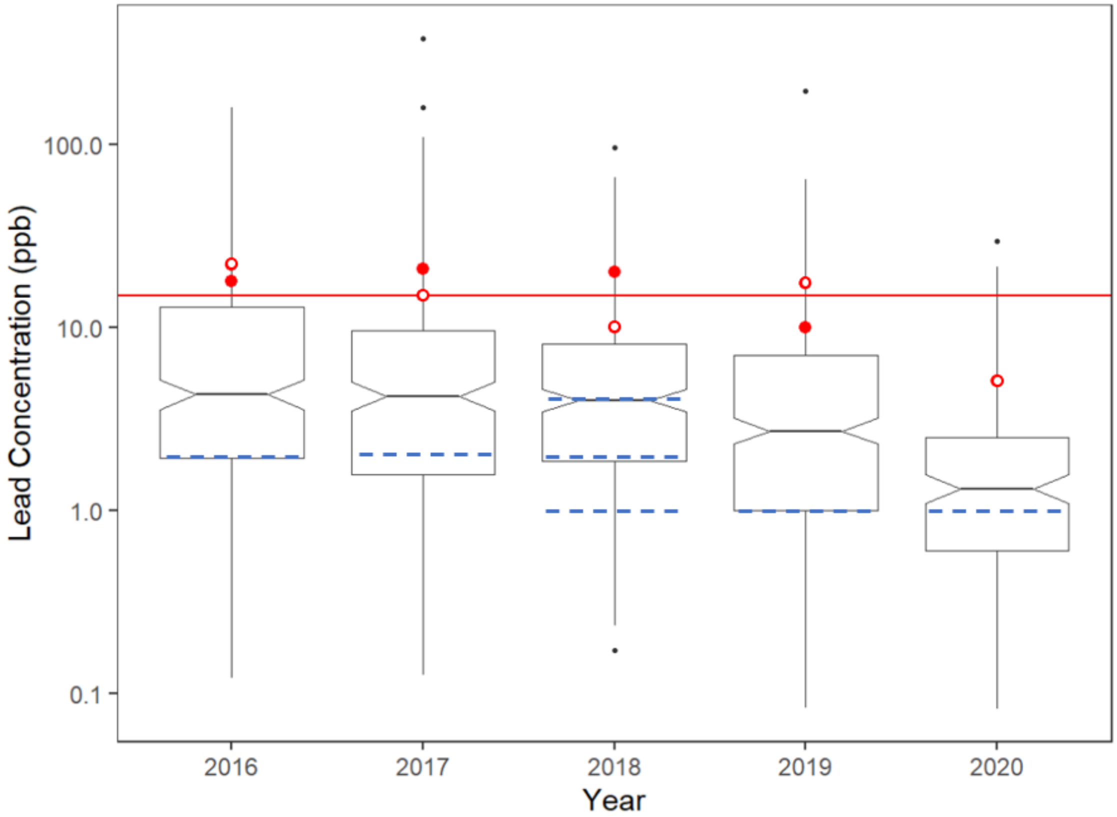

Figure S30: LCR compliance sampling data shown on a logarithmic concentration scale by year. The median is shown as the black bar within the box, encompassed by the 25th and 75th percentiles. The whiskers represent 1.5 times the interquartile range and all points outside of that range are represented by black dots. The notches indicate the confidence interval around the median. All censored values have been replaced with imputed estimates. The red dots indicate the corresponding 90th percentile concentration reported for each compliance period (two values per year, open circle reported in June and solid circle reported in December). The solid red line represents the EPA AL (15ppb) and the dashed blue lines within each of the box plots represent the reporting limits corresponding to each year of the data (**Reference SI Section II** for more information on reporting limits).

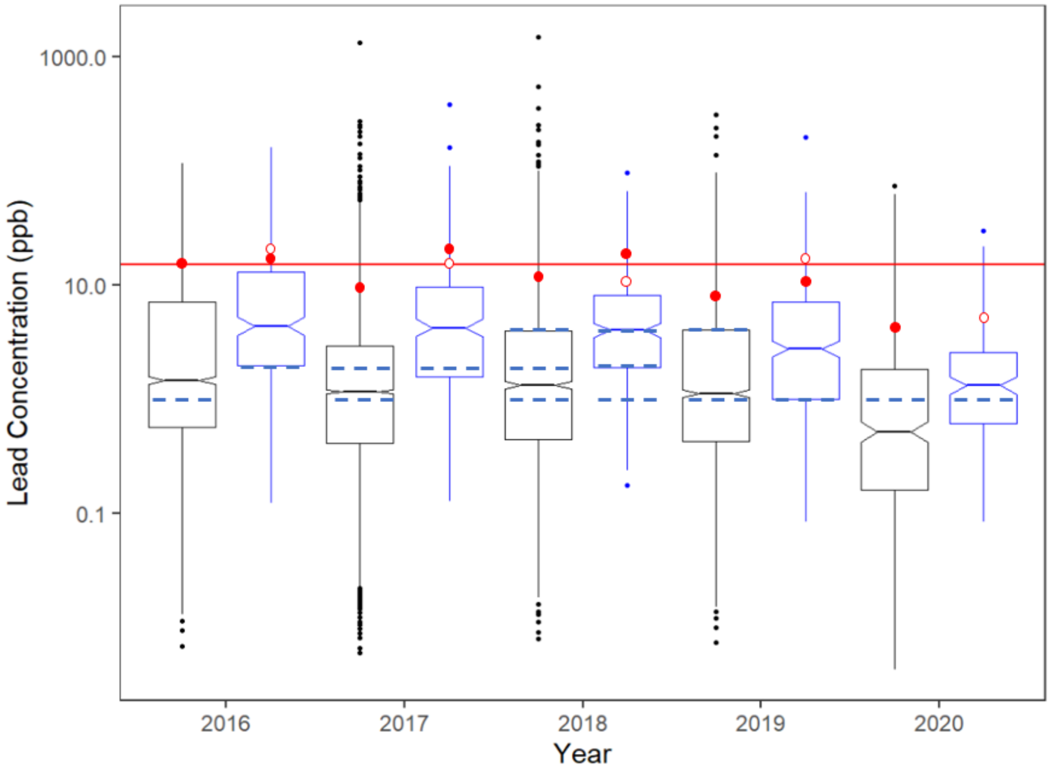

Figure S31: Notched boxplots of LCR (outlined in blue) and household (outlined in grey) lead concentration data shown on a logarithmic concentration scale by year. The median is shown as the solid line within the box, encompassed by the 25th and 75th percentiles. The whiskers represent 1.5 times the interquartile range and all points outside of that range are represented by blue dots. The red dots correspond to the 90th percentile lead concentration. For the LCR data, two dots are shown for data collected between 2016 and 2019, which correspond to reported LCR compliance lead concentration (open dots correspond to values reported in June, while filled dots correspond to values reported in December). The notches indicate the confidence interval around the median. All censored values have been replaced with imputed estimates. The solid red line indicates the EPA AL (15ppb), while the dashed blue lines indicate the reporting limits used by the different analytical laboratories for each year. For comparison, the customer-requested lead sampling data is shown for each year as well (in black) with corresponding detection limits provided as dashed blue lines within the boxplot. **SI Section II** provides additional information of reporting limits used in the customer-requested data.

Table S22: Summary statistics for LCR Compliance data by year

**Year**	**Sample size**	**Number of samples below reporting limit**	**Number of samples above AL**	**Reported 90th Percentile Value Reported by the utility (ppb) (June; December)**	**Arithmetic mean (ppb)**	**Median (ppb)**	**Standard deviation**
**2016**	259	96	41	**22; 18**	9.33	4.30	14.4
**2017**	246	84	26	**15; 21**	9.96	4.20	28.3
**2018**	267	125	27	10**; 20**	7.16	4.00	10.3
**2019**	345	104	26	**17.52;** 10	6.27	2.73	13.8
**2020**	158	63	2	5.1	2.23	1.32	3.35

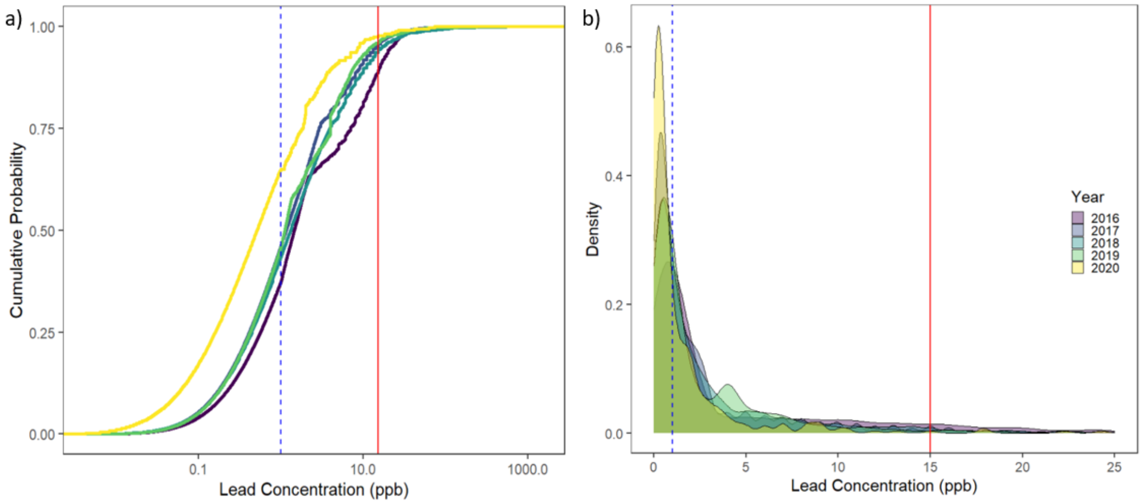

Figure S32: LCR compliance data by year presented as a) cumulative distribution plot on a logarithmic concentration scale and b) a density plot on an arithmetic scale. The solid red line represents the EPA AL and the dashed blue line represents a reporting limit of 1 ppb.

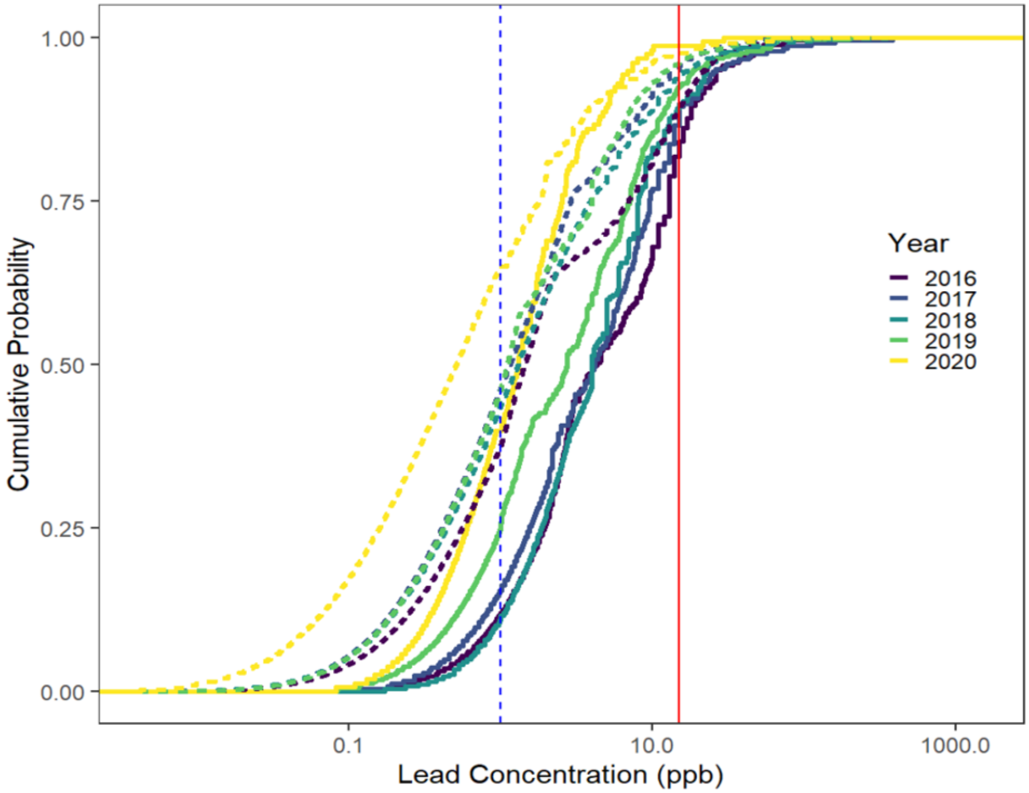

Figure S33: Cumulative distribution plot of lead concentrations from LCR data (solid lines) and customer-requested data (dashed lines) by year on a logarithmic concentration scale. The solid vertical red line represents the EPA AL of 15ppb. The dashed blue indicated the reporting limit of 1 ppb used in the LCR data.

C. *Pre- and Post-Orthophosphate*

Figure S34 shows the results of fitting two distributions to the customer-requested lead concentration data from samples taken after the implementation of orthophosphate for corrosion control. **Table S17** shows the associated statistical test results and mixture distribution parameters fit to the data. This analysis suggests that two types of households are in the system. When corrosion control is effective, households with lower lead concentrations can be represented by a distribution where the median and the 90th percentile are below detection, while the households with higher lead concentrations are represented by a distribution where the median is 3.3 ppb and the 90th percentile is 11.8 ppb.

The mixture weights indicate how many samples fall within each distribution: 60% of samples are attributed to the lower distribution and 40% of samples fall within the higher distribution. Since the lower household distribution has significantly lower lead concentrations than the compliance sampling, this suggests that most households are not Tier I (lead service line or lead plumbing in the home). This is consistent with data from the PWSA Lead Map, compiled from their lead service line inventory, showing 28% private side and 18% public side lead service lines in the distribution system.[16] Private side lead services lines are the portion of the service line that is owned by the homeowner, from the curb box to the home, while the public side lead service line is the portion owned and serviced by the utility, from the water main to the curb box.[17] The fraction of households with lead within indoor plumbing is more difficult to estimate, but housing stock age is predominantly pre-1979.

LCR compliance and customer requested data collected prior to and after the introduction of orthophosphate were compared. **Figure S35** provides a boxplot of the component distributions

from the mixture model fit to the customer requested data and the single lognormal distribution fit to the LCR data for both pre- and post-orthophosphate period delineated in **Table S23**.

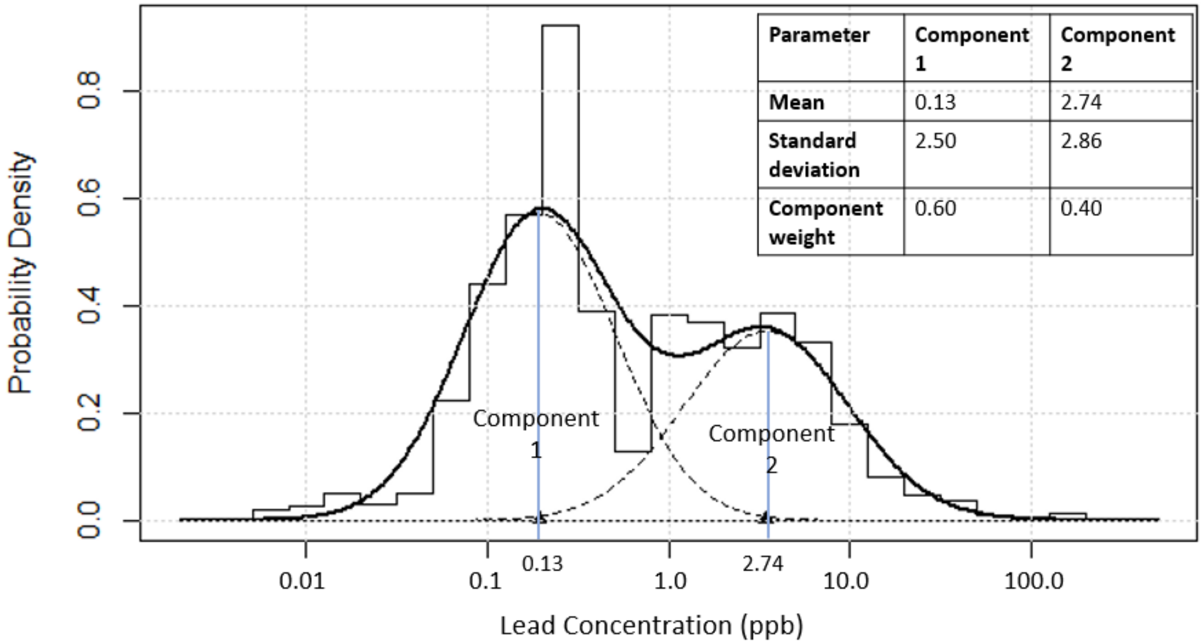

Figure S34: Probability density plot of Mixture distributions fit to post-orthophosphate introduction *Customer-requested* lead concentration data. The x-axis is the recorded lead concentrations in parts per billion on a logarithmic concentration scale and the y-axis is the probability density of each concentration. The solid black line provides a histogram of the original distribution, the dashed lines are the fitted distributions for components 1 and 2 and the bolded solid line is the combined fitted distribution. Mixture component parameters have been provided in the inset table and the means are provided as the blue vertical lines. Component 1 corresponds to the “lower” household distribution and Component 2 corresponds to the “higher” household distribution.

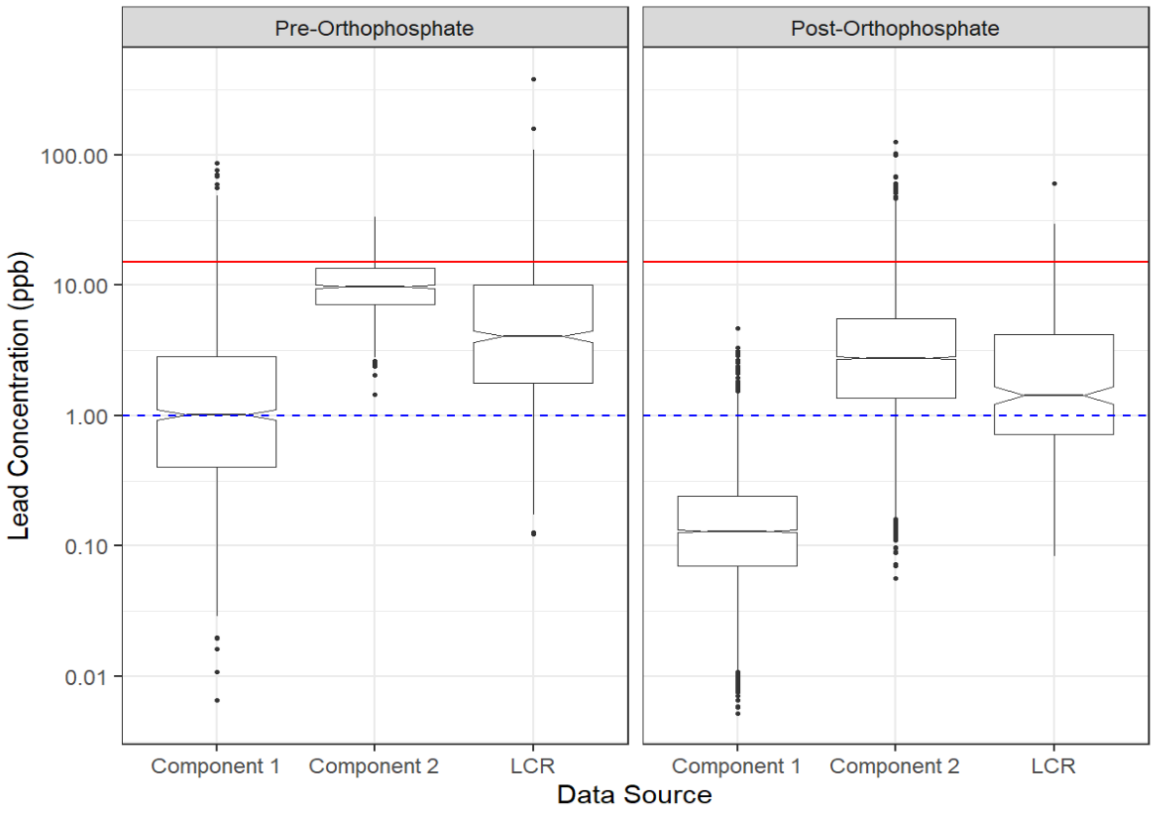

Figure S35: Boxplot of lead concentrations from the fitted LCR data and the mixture components characterizing the customer-requested data collected pre- and post-orthophosphate on a logarithmic concentration scale. The median is shown as the black bar within the box, which encompasses the 25th and 75th percentiles. The whiskers represent 1.5 times the interquartile range and all points outside of that range are represented by black dots. The notches indicate the confidence interval around the median. All censored values have been replaced with imputed estimates. The solid red horizontal line indicates the EPA AL (15 ppb). The dashed blue horizontal indicates the lowest reporting limit of 1 ppb used in the customer requested and LCR compliance data. Reference SI Section II for more information on reporting limits.

Table S23: Classification of lead sampling data for the pre- and post-orthophosphate analysis.

**Data Source**	**Pre-Orthophosphate**	**Excluded (Unclear or during transition)**	**Post-Orthophosphate**
LCR Data (reported every six months, no sample or analysis date)	June 2016, Dec 2016, June 2017, Dec 2017, June 2018, Dec 2018	June 2019	Dec 2019, June 2020
Household Sampling Data (continuously sampled since 2016; dates are analysis date, not sample date)	March 2016 – March 2019	April –May 2019	June 2019 – June 2020

D. *Monte Carlo simulations of the customer-requested data*

Figure S36 provides cumulative distribution plots of the simulated 90th percentile concentrations from the fitted customer requested mixture models by year. The red dots signify the corresponding reported 90th percentiles from the LCR compliance testing. The figure indicates that almost all of the simulated 90th percentile concentrations are less than the reported LCR compliance 90th percentile concentrations (the reported 90th percentile concentration in June 2018 was 10 ppb which was greater than 90.5% of the simulated concentrations and the reported 90th from June 2020 was 5.1 ppb which was greater than 94.5% of the simulated concentrations for that year). The reported 90th percentile in June 2020 is higher than most of the simulated concentrations. This is most likely a result of the ongoing optimization of the water distribution network which continues to bring lead concentration distributions lower over time. The optimization of corrosion control typically takes over a year, with lead concentrations continuously decreasing as dosing of orthophosphate is tuned to optimal levels.[18]

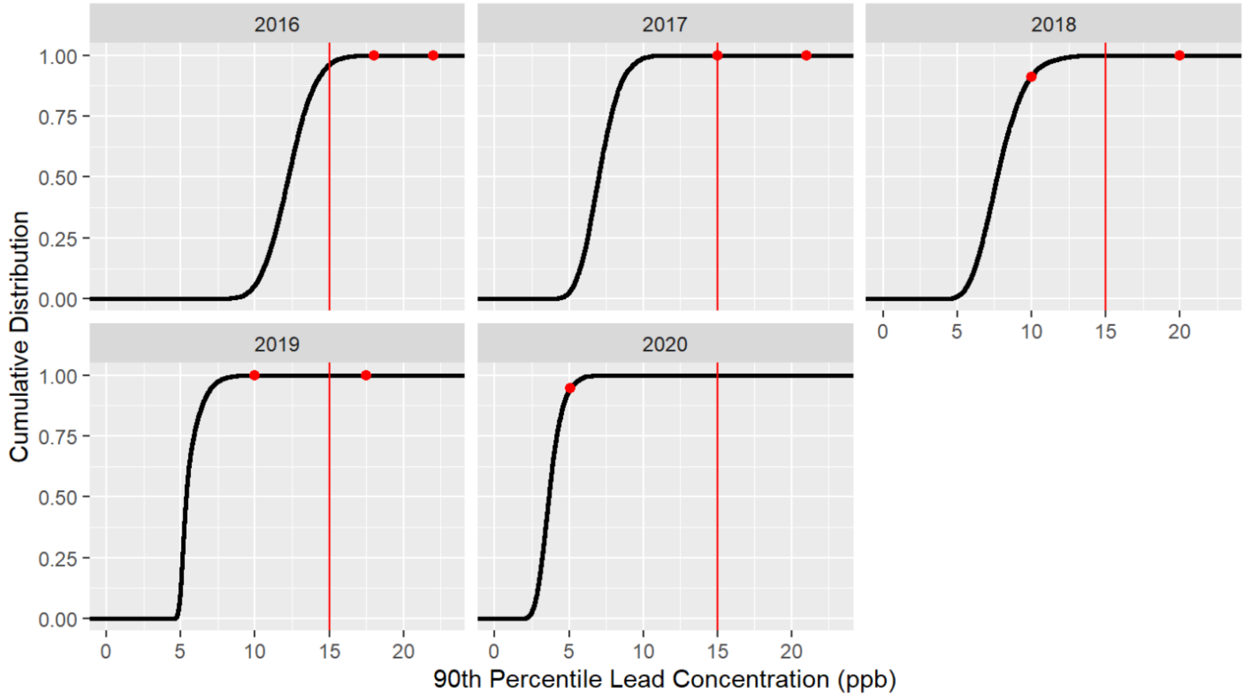

Figure S36: Cumulative distribution plots of the calculated 90th percentile concentrations from customer-requested Monte Carlo sampling pre- and post-orthophosphate. The solid red line indicates the EPA Action Limit of 15 ppb and the red dots indicate the corresponding LCR reported 90th percentile concentration per remediation stage.

E. *Spatial sampling bias assessment of customer-requested data*

Figure S37 provides a map of the fraction of samples above the reporting limit in each ZIP code within Pittsburgh's city limits. The map indicates significant spatial variability in the fraction of samples above the reporting limit with ZIP codes 15211, 15224 and 15217 exhibiting higher fractions of samples above the reporting limit than neighboring ZIP codes. It is important to note that ZIP codes 15226, 15216 and 15204 which show lower fractions of samples above the reporting limit are only partially within PWSA's service zone so fewer homes were sampled in this area, as shown in **Figure S38**. **Figure S38** shows significant variability in the fraction of homes sampled among ZIP codes with substantial sampling performed in ZIP code 15217 which was identified as having a higher fraction of samples above the reporting limit. It is important to note that sampling was concentrated in the western parts of the city as shown by the darker shade in ZIP codes 15201, 15206 and 15217. Other potential explanatory variables, including the prevalence of lead service lines, age of homes and household income should be explored to determine if they also influence sampling bias or the prevalence of elevated lead concentrations.

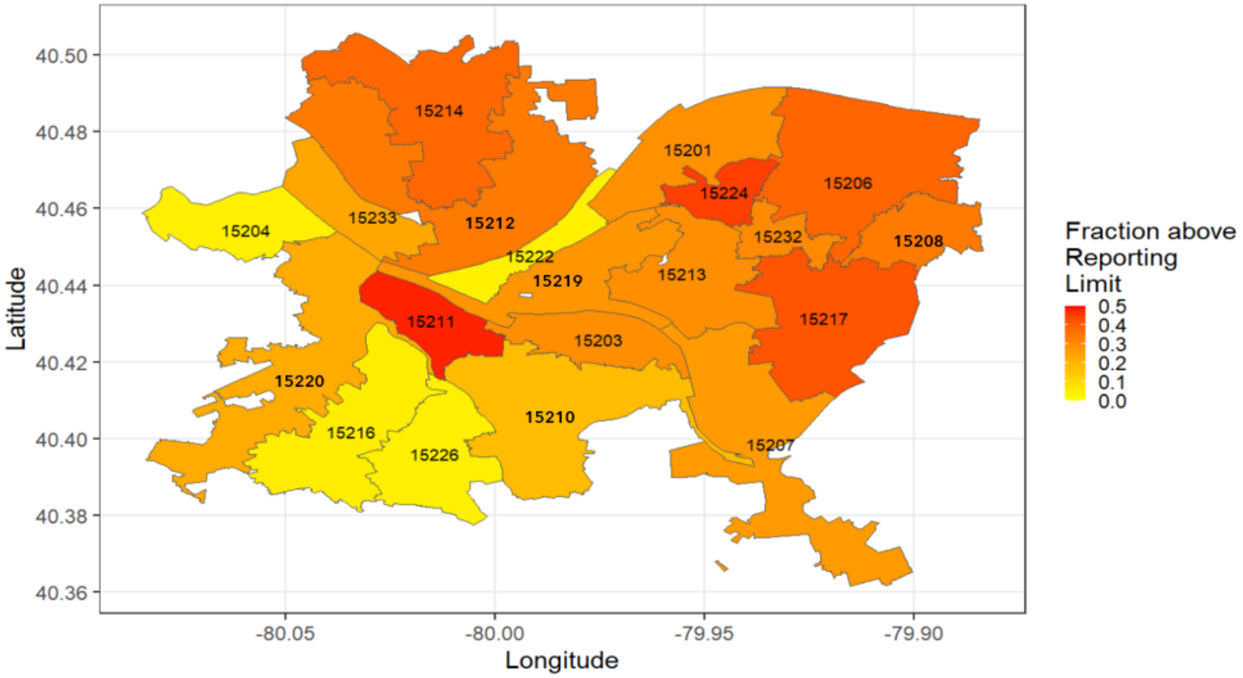

Figure S37: Map of the fraction of customer-requested samples above the reporting limit for each ZIP code within Pittsburgh's city limits.

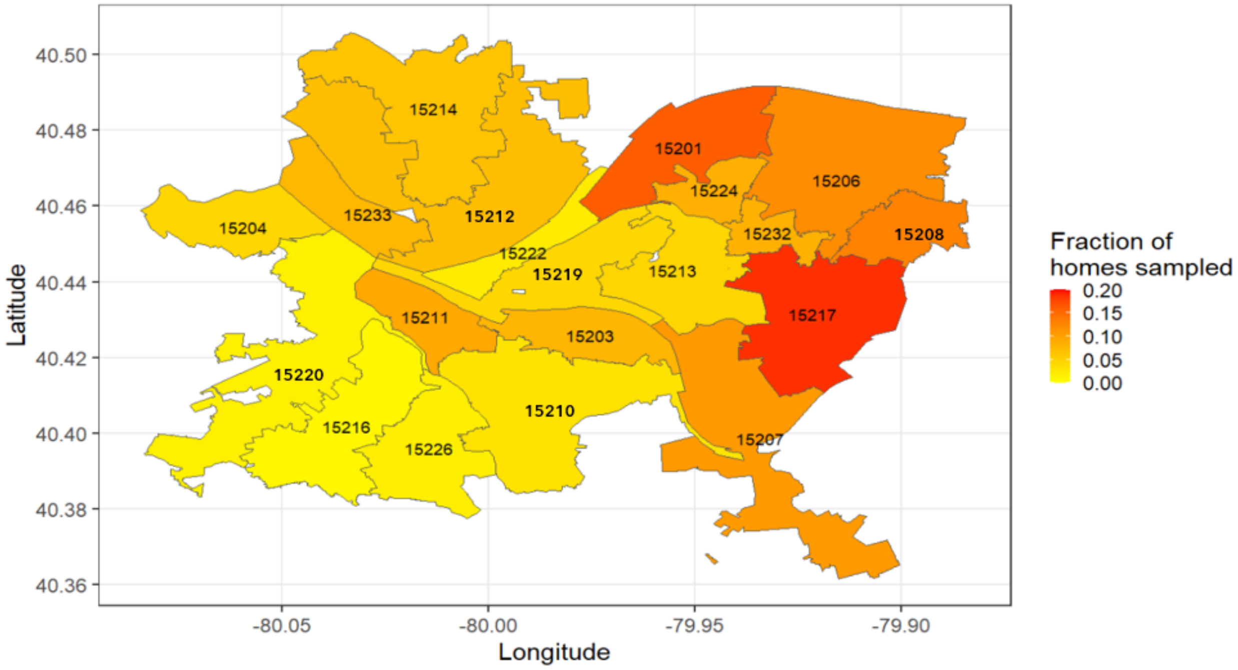

Figure S38: Map of the fraction of homes sampled through the customer-requested sampling program in each ZIP code.

References

(1) US EPA. Lead and Copper Rule, 1991.
(2) PWSA Prepares Water Distribution System for Orthophosphate. *Community Lead Response*, 2019.
(3) *DEP Cites Pittsburgh Water And Sewer Authority For*. Pennsylvania Pressroom. https://www.media.pa.gov:443/Pages/DEP_details.aspx?newsid=628 (accessed 2022-08-18).
(4) PWSA Continues Industry-Leading Lead Line Replacement Program. *Community Lead Response*, 2020.
(5) Blackhurst, M. Identifying Lead Service Lines with Field Tap Water Sampling. *ACS EST Water* **2021**, *1* (8), 1983–1991. https://doi.org/10.1021/acsestwater.1c00227.
(6) Customer Water Quality Data. *Community Lead Response*, 2021.
(7) Wilcoxon, F. Individual Comparisons by Ranking Methods. *Biom. Bull.* **1945**, *1* (6), 80–83. https://doi.org/10.2307/3001968.
(8) Massey, F. J. The Kolmogorov-Smirnov Test for Goodness of Fit. *J. Am. Stat. Assoc.* **1951**, *46* (253), 68–78. https://doi.org/10.1080/01621459.1951.10500769.
(9) Masters, S.; Welter, G. J.; Edwards, M. Seasonal Variations in Lead Release to Potable Water. *Environ. Sci. Technol.* **2016**, *50* (10), 5269–5277. https://doi.org/10.1021/acs.est.5b05060.
(10) Neath, A. A.; Cavanaugh, J. E. The Bayesian Information Criterion: Background, Derivation, and Applications. *WIREs Comput. Stat.* **2012**, *4* (2), 199–203. https://doi.org/10.1002/wics.199.
(11) Chung et al. - 2004 - Difficulties in Drawing Inferences With Finite-Mix.Pdf.
(12) *Allegheny County GIS Open Data*. https://openac-alcogis.opendata.arcgis.com/ (accessed 2022-05-31).
(13) *WPRDC • The Region's Open Data at Your Fingertips*. WPRDC. http://www.wprdc.org/ (accessed 2022-06-02).
(14) Bureau, U. C. *American Community Survey 5-Year Data (2009-2019)*. The United States Census Bureau. https://www.census.gov/data/developers/data-sets/acs-5year.html (accessed 2021-04-17).
(15) *PWSA's Community Lead Response Meets New Lead and Copper Rules Standards*. https://www.pgh2o.com/news-events/news/press-release/2020-12-24-pwsas-community-lead-response-meets-new-lead-and-copper (accessed 2022-08-03).
(16) Lead Map, 2021.
(17) Your Water Service Line. *Community Lead Response*.
(18) Cardew, P. T. Measuring the Benefit of Orthophosphate Treatment on Lead in Drinking Water. *J. Water Health* **2009**, *7* (1), 123–131. https://doi.org/10.2166/wh.2009.015.
